# Serum-free cultures of C2C12 cells show different muscle phenotypes which can be estimated by metabolic profiling

**DOI:** 10.1038/s41598-022-04804-z

**Published:** 2022-01-17

**Authors:** Mi Jang, Jana Scheffold, Lisa Marie Røst, Hyejeong Cheon, Per Bruheim

**Affiliations:** 1 Department of Biotechnology and Food Science, Norwegian University of Science and Technology, Hogskoleringen 1, 7491 Trondheim, Norway; 2 PoreLab, Department of Physics, Norwegian University of Science and Technology, Hogskoleringen 1, 7491 Trondheim, Norway

**Keywords:** Biochemistry, Cell biology

## Abstract

In vitro skeletal muscle cell production is emerging in the field of artificial lab-grown meat as alternative future food. Currently, there is an urgent paradigm shift towards a serum replacement culture system. Surprisingly, little is known about the impact of serum-free culture on skeletal muscle cells to date. Therefore, we performed metabolic profiling of the C2C12 myoblasts and myotubes in serum-free mediums (B27, AIM-V) and compared it with conventional serum supplementation culture. Furthermore, cell morphology, viability, and myogenic differentiation were observed for 7 days of cultivation. Intriguingly, the metabolic difference is more dominant between the cell status than medium effects. In addition, proliferative myoblast showed more distinct metabolic differences than differentiated myotubes in different culture conditions. The intracellular levels of GL3P and UDP-GlcNAc were significantly increased in myotubes versus myoblast. Non-essential amino acids and pyruvate reduction and transamination showed significant differences among serum, B27, and AIM-V cultures. Intracellular metabolite profiles indicated that C2C12 myotubes cultured in serum and B27 had predominant glycolytic and oxidative metabolism, respectively, indicating fast and slow types of muscle confirmed by MHC immunostaining. This work might be helpful to understand the altered metabolism of skeletal muscle cells in serum-free culture and contribute to future artificial meat research work.

## Introduction

The production of mammalian cells by in vitro cultivation techniques is of considerable importance not only in the biopharmaceutical industry for the production of therapeutic antibodies and vaccines in the bioprocess industry, but also cell therapy applications in the biomedical field^1,2^. Recently, in vitro cultured meat or artificial lab-grown meat is increasingly emerging as a novel concept in upcoming food science and technology^3^. However, current in vitro cell culturing methods still rely on animal-driven serum supplementation in culture medium, even though serum is controversial raw material. Serum, which is better known as fetal bovine serum (FBS), is mainly collected from the unborn calf through a crucial and undesirable way. In addition to these ethical problems, the use of animal-derived serums also causes serious scientific issues. Every batch of serum shows large variations and the composition of serum is not defined, resulting in phenotypical quantitative and qualitative discrepancy in in vitro cell production. A more serious risk are contaminations, such as viral, bacterial, and endotoxin infections derived from a host animal^4^. The presence of serum is a major obstacle for the further purification and validation of cell-based products^5,6^, and strongly suggests the replacement or no addition of serum in in vitro cell culture processes.

Although the field of the biopharmaceutical industry and clinical cell therapy application have long adopted serum-free culture model^6,7^, the field of artificial meat research, firstly introduced in 2013, still relies on serum supplementation^8,9^. Therefore, serum supplementation should be avoided and research direction should consider establishing serum-free systems in the field of artificial meat^10^. The majority of cells composing meat are skeletal muscle cells. Muscle stem cells proliferate as myoblasts and differentiate into myotubes by cell–cell fusion, forming multiple muscle fibers. Despite the importance of serum-free culture studies in artificial meat research, very few studies have explored the replacement of serum-free culture using primary myoblast from bovine, porcine, and murine origin^11–13^. Due to the difficulty of accessing primary cells, the C2C12, a mouse myoblast cell line, is often used in muscle cell research, including the field of artificial meat research^8,14^. Previously only a few research have shown the possibility of serum-free culture to cultivate C2C12 cells^15–17^.

Given that serum-free medium provides a completely new environment to the cells, it would be intriguing to investigate how serum-free environment affects the behavior of cells, from regulation of gene expression to the phenotype, compared to the conventional serum-supplemented medium. Previously, mouse embryonic stem cells in serum-free culture showed less variability of gene expression than serum-supplemented cultures based on single-cell transcriptomics analysis^18^. Moreover, Chinese hamster ovary (CHO) cells cultured in serum-free medium showed altered gene expression profiles in terms of nucleotide synthesis and lipid metabolism. Recently, the serum-free culture of *Drosophila* cells showed less oxidative stress and better retaining hemocyte-specific transcriptome profiles but limited metabolic alteration^19^. However, the metabolism of mammalian muscle cells in serum-free medium in comparison to serum-supplemented culture has not been elucidated yet.

Metabolomics is superior technology that provides a comprehensive profile of metabolites and is broadly acknowledged to be the omics discipline that is closest to the phenotype^20^. Therefore, metabolomics is considered the major technique for the characterization of meat composition and the exploration of biomarkers for quality control in the field of skeletal muscle research and the meat industry^21^. Previously, our group has reported the alteration of central carbon metabolism in *Bacillus subtilis* and *Saccharomyces cerevisiae* cultured in various mediums conditions and profiled intracellular metabolite pools in diverse human cell lines based on mass spectrometry targeted metabolomic technology^22,23^. Thus, we hypothesize that exploring metabolic pathways may provide information on the phenotype and overall biochemical mechanism of muscle cells influenced by diverse nutrient supplies, such as serum versus serum-free medium.

**Figure 1:**
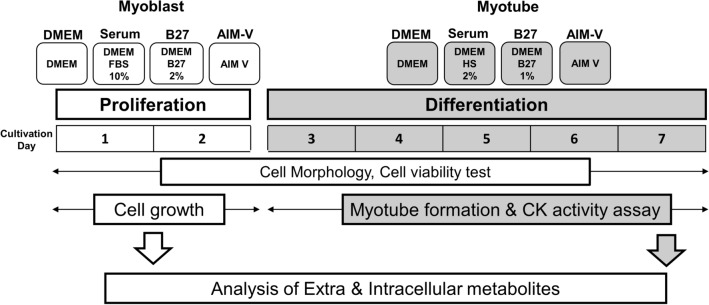
The experimental schematic workflow in this study. C2C12 cells were cultured for 7 days in the following 4 different types of mediums. (i) DMEM; only basic medium without any supplementation, (ii) Serum; DMEM supplemented with animal-derived serum. 10% of fetal bovine serum (FBS) and 2% of horse serum (HS) were used for proliferation and differentiation, respectively. (iii) B27; chemically defined serum substitute, 2% and 1% of B27 were supplemented in DMEM for proliferation and differentiation, respectively. (iv) AIM-V, chemically defined medium. Cell morphology and viability were monitored for the entire culture period. Cell growth was further profiled during the proliferation status, CK activity, myotube diameter, muscle contraction were further assessed for myogenic differentiation. Finally, culture samples from day 1 and day 7 representing myoblasts and myotubes were analyzed for the metabolomic study.

Therefore, we aim to investigate the metabolism of muscle cells using mouse myoblast C2C12 cell line in serum-free culture models. C2C12 cells were cultured in 4 different mediums: (i) only DMEM medium without any supplementation, (ii) DMEM containing animal-derived serum, (iii) DMEM including the serum substitute B27, (iv) chemically defined medium AIM-V. During the entire cultivation period, cell viability and morphology were monitored. In addition, the myoblast growth profile and the creatine kinase activity for the assessment of myogenic differentiation were further evaluated. Finally, the extracellular and intracellular metabolites of myoblast and myotubes are analyzed based on a targeted metabolomics approach (Fig. 1). Our work is of benefit to understand the behavior of the muscle cell in serum-free culture and contribute to artificial meat research.

## Result and discussion

### C2C12 cells successfully proliferated and differentiated into myotubes in serum-free cultures for 7 days

**Figure 2:**
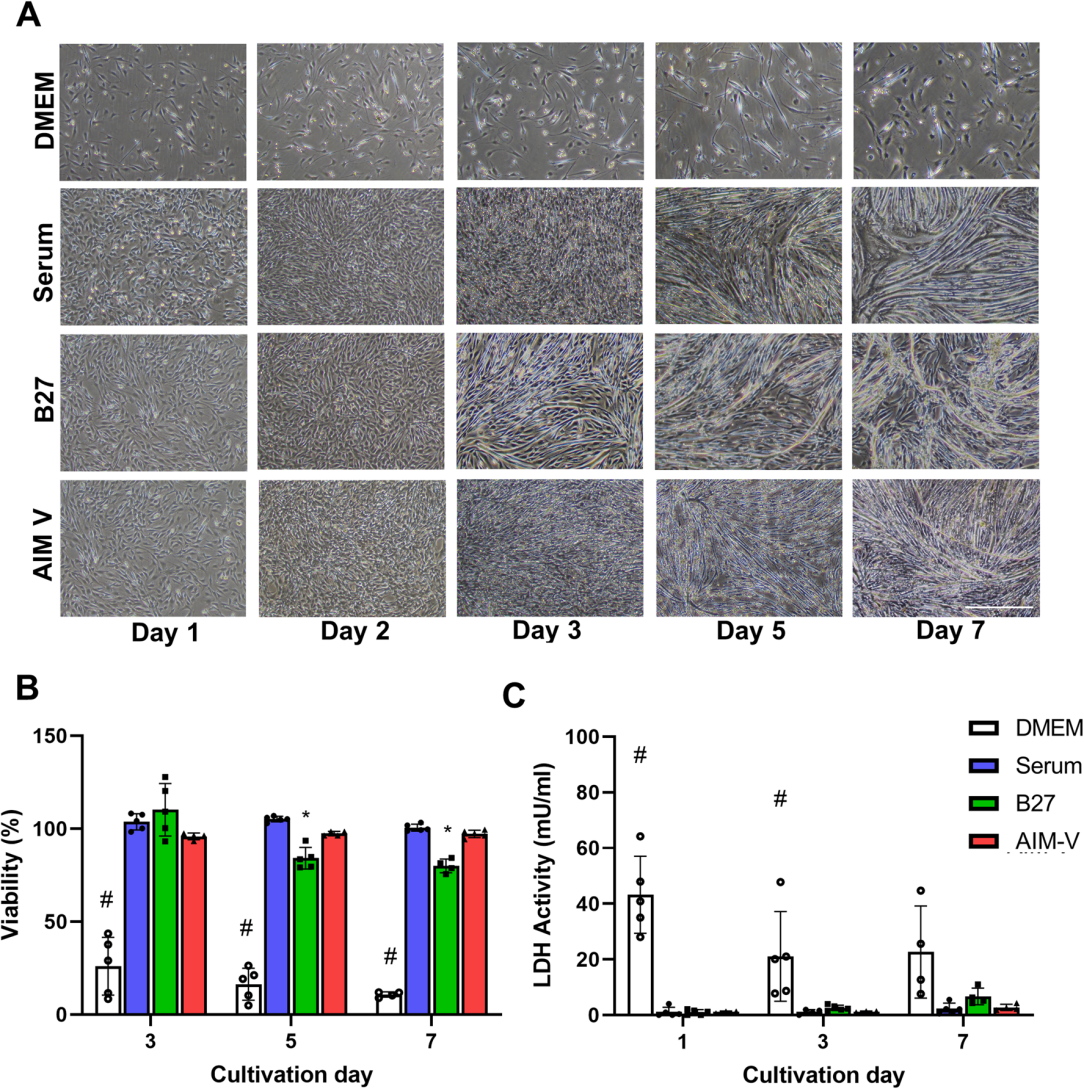
(**A**) Phase-contrast images of cell morphology during 7 days of cultivation in the different mediums. Myoblasts proliferation until the 2nd day of cultivation then myogenic differentiation was initiated from the 3rd day of cultivation. Fully differentiated myotubes were formed on 7th day of cultivation. Scale bar indicates 500 µm. (**B**) Cell viability based on Prestoblue assay was performed for 7 days of cultivation. Cell viability was expressed as a relative change on day 1 of each condition. (**C**) LDH activity in the medium was measured for 7 days. (n) = 3–5 in each group. One-way ANOVA followed by Tukey’s test was performed for the multiple comparisons. *p < 0.05 versus Serum and AIM-V, #p < 0.05 versus Serum, B27, and AIM-V at each cultivation day. Data presented as average ± standard deviation (SD).

Cell morphology was observed over 1 week of cultivation in 4 different types of mediums to monitor cellular proliferation and differentiation status. Skeletal muscle cells differentiated into multinucleated myotubes, resulting in the formation of myofibers^24^. Representative phase-contrast images from each day of cultivation are shown in Fig. 2A. During the proliferation period, C2C12 cells showed the bipolar shape of myoblast at day 1 and further proliferated until day 2, when they reached full confluency in serum, AIM-V, B27 medium. In the case of only DMEM culture, cells did not proliferate at all during the whole cultivation period, showing a single spindle bundle or a round shape. Considering the myogenic differentiation period from 3rd to 7th day of culture, cells apparently started fusing from day 3 and were completely differentiated into myotubes at the 7th day of cultivation in all culture mediums except DMEM. Interestingly, C2C12 cultured in B27 formed myotubes earlier (day 5) than cells cultured in serum and AIM-V medium, showing randomly aligned myotubes including few clusters at day 7. In contrast to B27, serum and AIM-V cultures resulted in straight and unidirectional myotubes at day 7. Overall, distinct and different myotube morphology was observed in the 3 different mediums.

After confirming that proliferation and differentiation can be achieved in the three different mediums except DMEM, we further assessed the cell viability and cell death during a 7-day cultivation period (Fig. 2B,C). Prestoblue, a cell-permeable dye, was used for the assessment of cell viability. In addition, LDH activity in the medium was further determined as it not only can be complementary to cell viability but also is a non-invasive method. This is because only membrane-damaged cells release LDH into the medium, assuming cell death^25^. DMEM culture showed significantly decreased cell viability and elevated LDH activity compared to the 3 other medium cultures, indicating extra supplementation to the basic medium is necessary to support the survival of cells. C2C12 cultured in serum and AIM-V showed constant high cell viability (> 90%) and the lowest LDH activity throughout the entire cultivation period. B27 culture showed significantly decreased cell viability, but still more than 80%, while LDH activity was not significantly different compared to serum and AIM-V, this might be due to the cell clumping. Altogether, overall cell viability was higher than 80% and AIM-V might offer less stressful environmental conditions for the C2C12 cells than the B27 serum substitute during the entire cultivation period.

### Different growth and differentiation profiles were observed among Serum, B27, and AIM-V cultures

**Figure 3:**
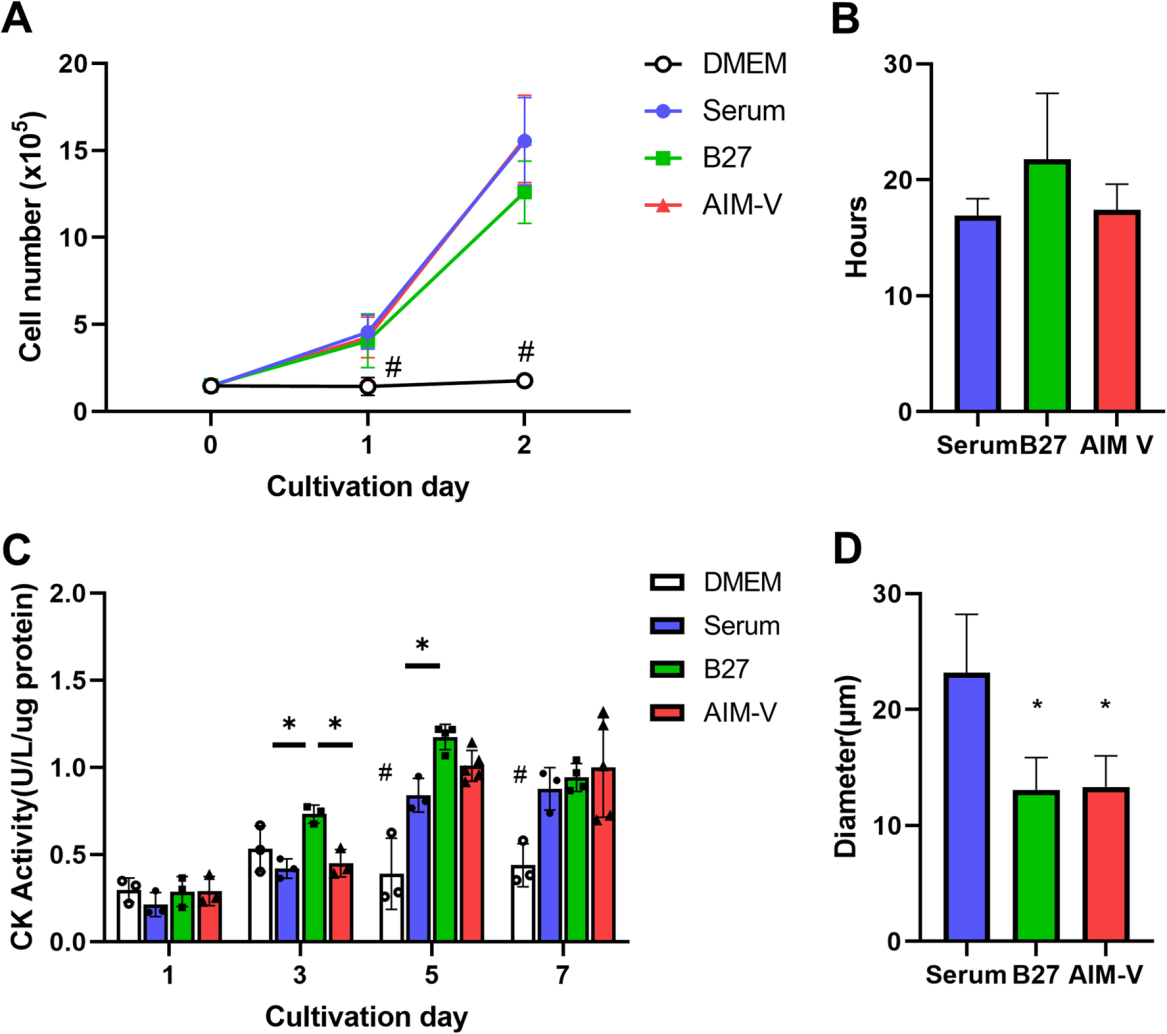
Cell growth profiles during from day 0 to 2 of cultivation. (**A**) The growth rate for 2 days of cultivation. (n) = 3, #p < 0.05 versus Serum, B27, and AIM-V at each cultivation day. (**B**) Doubling time in each culture. (n) = 3 (**C**) Creatine kinase (CK) activity was measured for the assessment of myogenic differentiation during the entire cultivation period. (n) = 3–5. One-way ANOVA followed by Tukey’s test was performed for the multiple comparisons. *p < 0.05, #p < 0.05 versus Serum, B27, and AIM-V at each cultivation day. (**D**) Diameter of myotubes at day 7. In total, 20 randomly chosen myotubes were measured in each culture. *p < 0.05 versus Serum. Data presented as average ± standard deviation (SD).

Growth profiles in the proliferative myoblast were further investigated to determine growth rates and doubling times (Fig. 3A,B) until the second day of cultivation. C2C12 cells cultured in serum and AIM-V showed a higher growth rates trend compared to B27 cultures. However, such growth rates between Serum, B27, and AIM-V were not significant at day 2. Since DMEM culture does not support proliferation at all, we only calculated the doubling time in 3 culture conditions. Serum and AIM-V cultures showed similar doubling time, 15.7 ± 1.5 h and 16 ± 2 h, respectively, whilst B27 culture showed higher doubling time (20 ± 4 h), indicating that AIM-V medium might better support the growth than B27. However, our data showed no statistically significant difference between the three culture models. As the doubling time is lower than 20 h, we decided to collect cells at day 1 (24 h) for the metabolomic study to further investigate the myoblast phenotype. Furthermore, we evaluated myogenic differentiation by measuring creatine kinase (CK) activity^26^ throughout the entire cultivation (Fig. 3C). Except for DMEM, cells cultured in Serum, B27, and AIM-V culture gradually had increased CK activity after 3 days and reached the highest activity on day 7. Interestingly, B27 culture showed the fastest maturation of myotubes, and AIM-V culture showed a similar increasing CK activity rate compared to Serum culture. At the end of cultivation, the CK activity was similar in Serum, B27, AIM-V culture, with no statistically significant difference. Moreover, we measured the diameter (Fig. 3D) of myotubes from morphology images and observed muscle twitching at day 7 (supplementation video) in all cultures except DMEM. While the myotubes in Serum culture presented the largest diameter (23 ± 5 µm), those of AIM-V and B27 had smaller diameters (13 ± 2 µm). All myotubes showed contraction movement at day 7, confirming muscle cell differentiation. Myotubes grown in B27 showed different patterns of muscle twitching compared to Serum and AIM-V culture, which was confirmed by video analysis (Supplementary Fig. 4). Specifically, a higher time-to-single twitching and lower frequency of movement in B27 culture were shown than in Serum and AIM-V cultures (Supplementary Fig. 4B). Furthermore, the strongest movement of twitching was observed in larger areas in B27 culture, whereas weaker movement of twitching was determined only in smaller areas in Serum and AIM-V (Supplementary Fig. 4C). Interestingly, the characterization of diameter and contraction of myotubes could be classified into slow or fast types of muscle^27^. Since thinner diameter and slower contraction speed were shown in B27 culture, suggesting that slow type of muscles might be more dominant in B27 than in Serum culture. Taken together, myotubes in all three cultures at day 7 showed similar CK activity, but different sizes and twitching modes were observed among Serum, B27, and AIM-V cultures. Therefore, we decided to collect cells on day 7 to represent myotube status for further metabolomics study.

### Significant differences in the rate of glucose and glutamine consumption and lactate production between serum-free and serum-supplemented cultures

**Figure 4:**
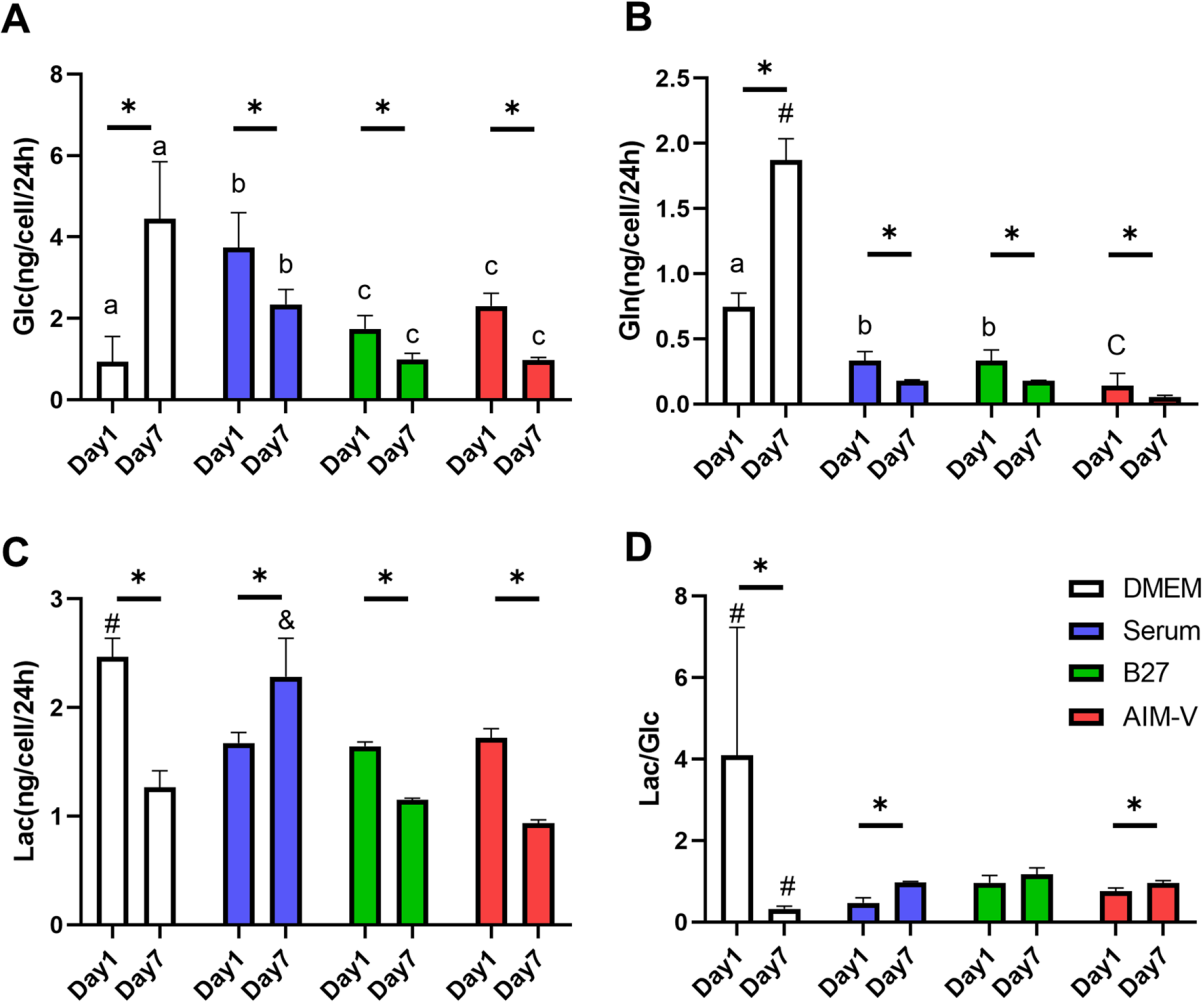
The rates of consumption and secretion of extracellular metabolites at day 1 (myoblasts) and day 7 (myotubes) in DMEM, Serum, B27, and AIM-V cultures. The medium was collected from day 0 to 1 and 6 to 7, representing day 1 and 7, respectively. (**A**) The rate of glucose consumption. (**B**) The rate of glutamine consumption. (**C**) The rate of lactate secretion. (**D**) The ratio of lactate secretion to glucose production. (n) = 4. The student’s t-test was performed to compare day 1 and day 7 in each different culture. *p < 0.05. One-way ANOVA followed by Tukey’s test was performed for the multiple comparisons. #p < 0.05 versus Serum, B27, and AIM-V at each cultivation day. &p < 0.05 versus DMEM, B27, and AIM-V at each cultivation day. a–c, different letters in the same day indicate the significant statistical difference. Data presented as average ± standard deviation (SD).

The difference in the rate of extracellular glucose and glutamine consumption and secretion of lactate from myoblasts (day 1) and myotubes (day 7) was compared in DMEM, Serum, B27, AIM-V cultures (Fig. 4). DMEM culture showed significantly different consumption and secretion trends compared to all others on day 1 and 7. Even though the absolute rates of consumed glucose and glutamine, and secreted lactate are different, the significant decreasing trend of glucose and glutamine consumption was shown between day 1 and day 7 in Serum, B27, and AIM-V cultures, indicating proliferative cells require uptake of carbon and nitrogen sources. Overall, serum-free cultures consumed less glucose and glutamine compared to serum culture (Fig. 4A,B). Interestingly, the secretion of lactate was shown to be different between serum and serum-free cultures. For example, the lactate secretion rates are similar between all cultures at day 1 (1.68 ng/24 h/cell). Unlike serum culture, the only serum-free cultures showed a lower secretion of lactate at day 7 compared to day 1 (Fig. 4C). Since lactate can cause the alteration of acidity and osmolality in the medium^28^, less accumulation of lactate (0.5 fold change compared to serum culture) in the medium can be an advantage for serum-free cultures. We further calculated the ratio of consumed glucose to secreted lactate to know the rate of secretion versus consumption (Fig. 4D). Surprisingly, all cultures showed the consistent trend that the ratio is higher in myotubes (day 7) than in myoblasts (day 1), except for DMEM culture. In addition, the ratio was shown to be the same in Serum and AIM-V, 0.97 ± 0.02 and 0.96 ± 0.05, respectively. Since the measurement of glucose consumption and lactate secretion from the medium is non-invasive, tracing altered consumption and secretion rates could provide additional information for the assessment of the maturation of skeletal muscle cells.

### Metabolite profiles are more dependent on growth status than medium conditions

Next, we analyzed intracellular metabolites involved in central carbon metabolism, such as the intermediates of glycolysis, TCA cycle, and pentose phosphate pathway (PPP), nucleotide phosphates, other sugar phosphates, deoxynucleotide phosphates, amino acids, and lactate. In total, 63 intracellular metabolites were quantified. To validate the quality of the sampling and quenching procedure, the adenyl energy charge (AEC), reflecting an index used to measure the energy status of biological cells, was calculated from the respective intracellular concentration of AMP, ADP, and ATP. The energy charge in physiological cells is reported to range from 0.7 to 0.95^22,29^. Our experimental AEC was between 0.77 and 0.82, indicating that the metabolite sampling procedure was acceptable (Supplementary Fig. 3).

**Figure 5:**
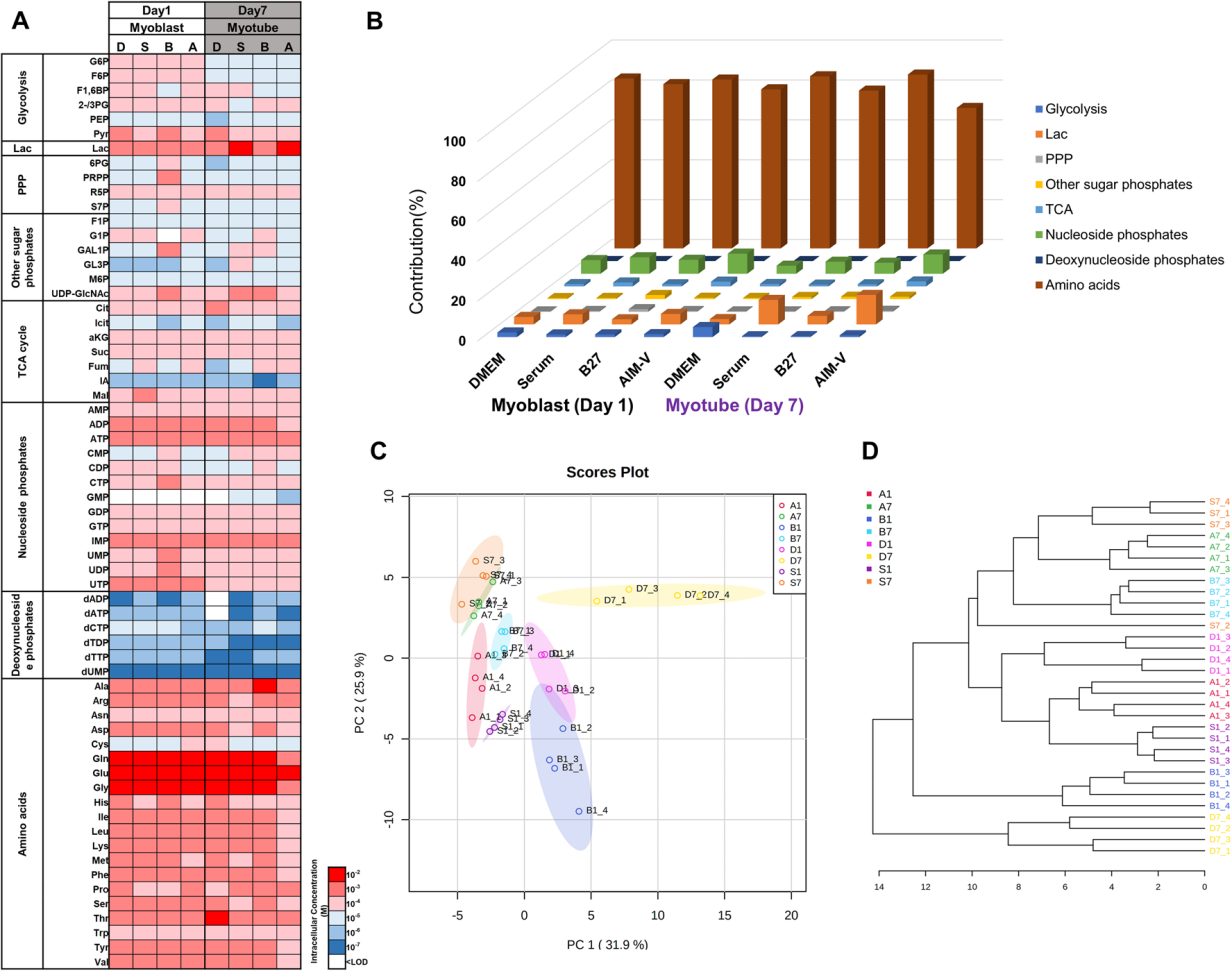
Overview of intracellular metabolites at day 1 (myoblasts) and day 7 (myotubes) in DMEM, Serum, B27, and AIM-V cultures. (**A**) Heat map of the average order of magnitude of intracellular concentrations (M). D, S, B, and A indicate DMEM, Serum, B27, and AIM-V, respectively. (**B**) Contribution (%) of metabolite classes to the total measured levels of metabolites. (**C**) Score plot of Principal component analysis (PCA). (**D**) Hierarchical clustering analysis (HCA) based on average distance and Ward clustering algorithm was performed on Metaboanalyst. A1, B1, D1, and S1 indicate AIM-V, B27, DMEM, and Serum culture at day 1 (myoblasts), and A7, B7, D7, and S7 indicate AIM-V, B27, DMEM, and Serum culture at day 7 (myotubes). (n) = 4.

The intracellular metabolites levels varied in the range of micromolar to millimolar concentrations (Fig. 5A). Amino acids were the most abundant metabolites, whilst deoxynucleotide phosphates had the lowest concentration in all culture conditions. Intriguingly, C2C12 cells cultured in B27 showed significantly higher levels of pentose phosphate metabolites (R5P, PRPP) and pyrimidine nucleotides (UMP, UDP, UTP, CTP) compared to other cultures at day 1.

However, the distribution among metabolite classes showed a more considerable divergence between myoblast (day 1) and myotube (day 7) in the different mediums (Fig. 5B). A significant increase in lactate pool was observed in myotube compared to myoblast except for DMEM. The AIM-V and Serum cultures presented a similar metabolic shifting in terms of significantly decreased contribution of glycolysis and pentose phosphate pathway (PPP)-related metabolites, nucleoside phosphates, and amino acids (decreased trend but not significant in Serum) from proliferating myoblasts to differentiated myotubes. This might be due to the fact that differentiated cells might shift their biosynthetic rate since they stop proliferating, which might be evidenced by low consumption rates of extracellular glucose and glutamine per cell (Fig. 4A,B). Previously, significantly downregulated mRNA expression of hexokinase (*HK*), aldolase (*ALDO*), pyruvate kinase (*PK*) in myotubes versus myoblasts were reported^30^. Consistent results previously reported that glycolysis occurs in proliferating C2C12 myoblasts, which later is followed by a differentiation process accompanied by autophagy and mitophagy, resulting in increasing oxidative phosphorylation activity^31,32^. Furthermore, an overall downregulated gene expression regulating nucleic acid and protein metabolism was observed during differentiation^31,33^. The pentose phosphate pathway (PPP) is upregulated in pathophysiological conditions and is known to be inactive in muscle cells^34^. Our metabolite profile results also showed significant downregulated PPP after myogenic differentiation. Importantly, intracellular lactate contribution is dramatically increased after differentiation.

To determine an overview of clustering, unsupervised principal component analysis (PCA) and hierarchical cluster analysis (HCA) were performed. The PCA score plot indicated that only DMEM culture at day 7 had a distinct altered intracellular metabolic profile compared to all other cultures. All myotube cultures (A7, B7, S7) were separated from myoblast cultures along PC2 and clustered together, indicating that metabolic difference is more dominant between the cellular status (proliferation vs. differentiation) than between medium conditions (Serum, B27, AIM-V) (Fig. 5C).

We further performed HCA to confirm the outcome of the PCA analysis (Fig. 5D). D7 is isolated from all groups followed by the B1 group showing the high concentration of pyrimidine nucleotides and pentose phosphate pathway metabolites. This might be due to the presence of galactose in B27. Galactose can enter the PPP at various stages and it can be further converted into G6P which is a substrate for PPP. Furthermore, galactose-driven glucose is shuttled into the PPP rather than glycolysis^35^. It has been confirmed by the manufacturer that AIM-V does not contain any galactose. Surprisingly, AIM-V and Serum cultures are clustered together at day 1 (myoblasts) and day 7 (myotubes). One Serum replicate was clustered with B27 at day 7. Taken together, the PCA and HCA analyses showed that C2C12 cells cultured in AIM-V had a more similar metabolic phenotype compared to Serum culture than B27. Furthermore, the metabolic difference is greater between cell status than extracellular nutrient composition.

### Intracellular GL3P and UDP-GlcNAc levels are significantly increased in myotube compared to myoblast in Serum, B27, and AIM-V cultures

**Figure 6:**
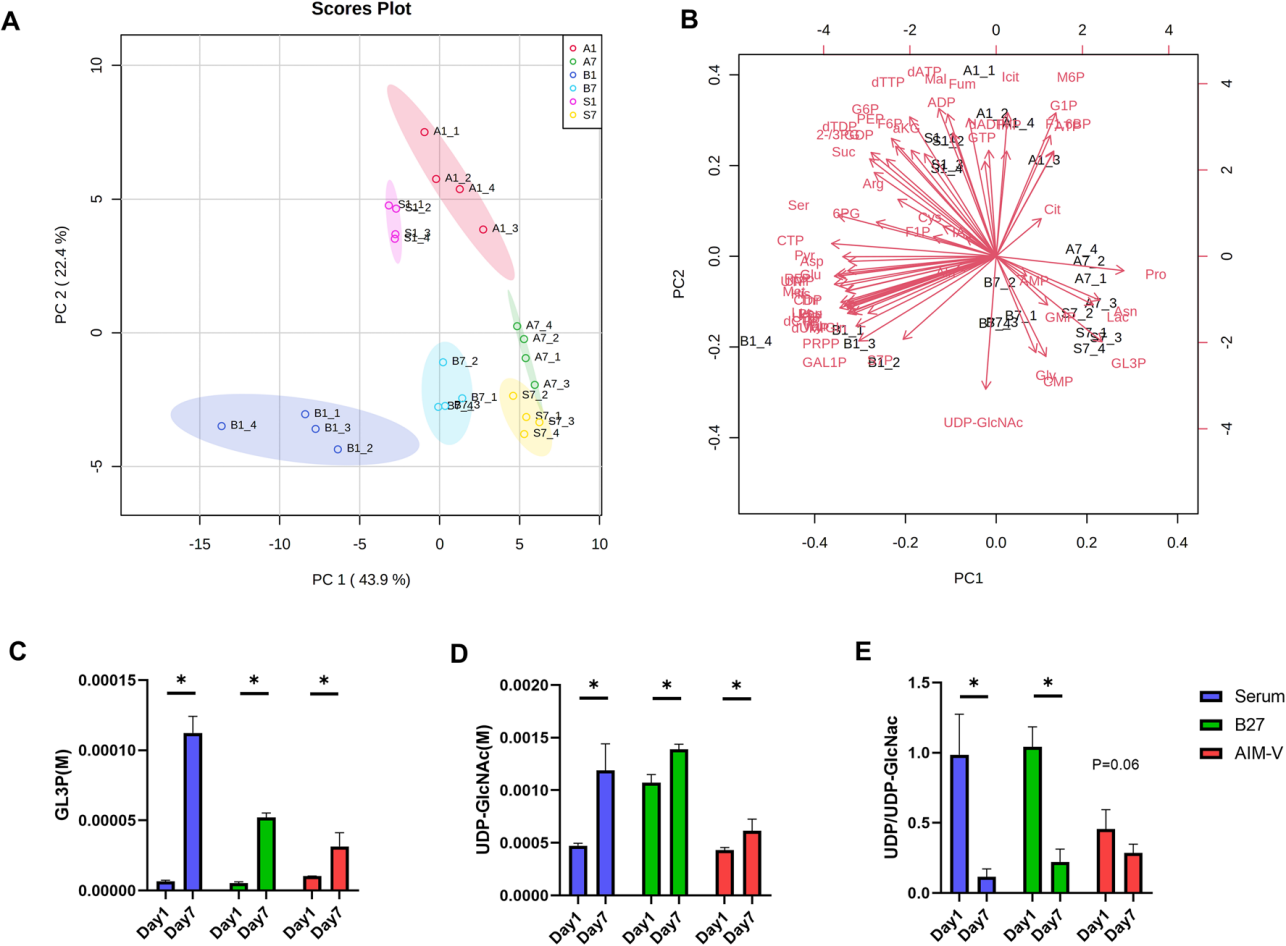
Principle component analysis (PCA) of intracellular metabolites datasets of C2C12 myoblasts (day 1) and myotubes (day 7) cultured in Serum, B27, and AIM-V. (**A**) Score plot of PC2 versus PC1. The individual shaded area presents the 95% confidence interval of the group. (**B**) Biplot. In total, 66% of the data variation is explained by PC1 and PC2. A1, B1, and S1 indicate AIM-V, B27, and Serum culture at day 1, and A7, B7, and S7 indicate AIM-V, B27, and Serum culture at day 7. Significantly increased metabolites in myotubes (day 7) versus myoblasts (day 1) cultured in Serum, B27, and AIM-V mediums. (**C**) Absolute intracellular concentration (M) of GL3P. (**D**) Absolute intracellular concentration (M) of UDP-GlcNAc in Serum, B27, and AIM-V culture. (**E**) The ratio of UDP/UDP-GlcNAc, indicating O-GlcNAcylation. (n) = 4. The student’s t-test was performed to compare day 1 and day 7 in each different culture. *p < 0.05. Data presented as average ± standard deviation (SD).

Next, we further explored how the intracellular metabolism alters between myoblasts (day 1) and myotubes (day 7) in Serum, B27, AIM-V except for DMEM medium. An unsupervised PCA was performed to visualize the overview of the normalized intracellular metabolites data set. There is a clearer classification between proliferating myoblast (A1, B1, S1) versus differentiated myotube (A7, B7, S7) than the culture medium factors. Surprisingly, cells in the proliferative status (A1, B1, S1) showed more dramatic metabolic differences than differentiated myotubes cultured in 3 different mediums in the PCA score plot (Fig. 6A). Myotubes cultured in all 3 mediums all tend to converge together, indicating that the variation of metabolic phenotypes in proliferating myoblast is larger than in differentiated myotube. Metabolic regulation in proliferating cells might be more sensitively influenced by the extracellular environment than in differentiated cells. A similar phenomenon was observed in proliferative intestinal epithelial cells that they are more sensitive than differentiated cells in response to extracellular mycotoxin treatment, which was confirmed by transcriptome analysis^36^.

Regarding the comparison of 3 different mediums, myoblast cultured in B27 still showed a dramatic difference compared to Serum culture, even though they shared the same basic medium (DMEM). AIM-V and Serum cultures are clustered together in the PCA score plot, which is consistent with previous results (Fig. 5C).

We further explored which metabolites are differently distributed between myoblast and myotube. In the PCA biplot, most metabolites contributed to the myoblast group at day 1 (Fig. 6B), which is in line with the above-mentioned results. Only Lac, CMP, Pro, Asn, Gly, UDP-GlcNAc, and Glycerol-3-phosphate (GL3P) were upregulated in the myotube group (A7, B7, S7). Among them, GL3P and UDP-GlcNAc showed significantly elevated concentrations between myotube versus myoblast in all 3 groups (Fig. 6C,D). GL3P stands at the crossroad of glucose, lipid, and energy metabolism in mammalian cells and participates as an electron transfer shuttle to mitochondria, but is also incorporated into the glycerol lipid and fatty acid cycle^37^. Previously, high production of GL3P was observed in intensive flight insect muscles^38^. Glycerol-3-phosphate dehydrogenase (GPDH) reversibly catalyzes the conversion of dihydroxyacetone phosphate to GL3P. Furthermore, mitochondrial glycerol 3-phosphate dehydrogenase (mGPDH) has been identified as an important regulator for muscle differentiation and regeneration and authors reported that the knockdown of mGPDH significantly downregulated myogenic differentiation^39^. Therefore, the GL3P shuttle might be a key target to regulate the maturation of skeletal muscle differentiation.

UDP-GlcNAc is a final product from the hexosamine biosynthetic pathway and serves as the donor substrate for O-GlcNAcylation which is the post-translational modification process that regulates fundamental cellular processes. Only a few studies have reported the regulation process of the skeletal muscle by O-GlcNAcylation to date. O-GlcNAcylation transferase (OGT) uses UDP-GlcNAc as a substrate to trigger O-GlcNAcylation process followed by a release of UDP^40^. Knockdown of OGT resulted in the accumulation of intracellular UDP-GlcNAc in muscle tissue^41^. Previously, negative regulation of O-GlcNAcylation was observed during terminal myogenic differentiation in C2C12 cells and the authors concluded that decreased O-GlcNAcylation may have a common role in the differentiation of cells of muscle lineage^42^. In addition, the complete ablation of OGT in muscle satellite cells in mice resulted in impaired proliferation, indicating O-GlcNAcylation is required for supporting proliferation^43^. We further calculated the ratio of UPD-GlcNAc to UDP to present O-GlcNAcylation (Fig. 6E). The fold change from myoblast to myotube was 0.12, 0.21, 0.65 in Serum, B27, AIM-V culture, respectively, which indicates decreased O-GlcNAcylation.

Taken together, to the best of our knowledge, this is the first study to provide solid evidence that intracellular GL3P and UDP-GlcNAc levels were significantly upregulated in differentiated skeletal muscle, suggesting it as a tool to evaluate the maturation of the skeletal muscle and that it offers a potential target for regulation of skeletal muscle generation.

### Not only non-essential amino acids but also the reduction and transamination of pyruvate are different between Serum, B27, and AIM-V cultures

**Figure 7:**
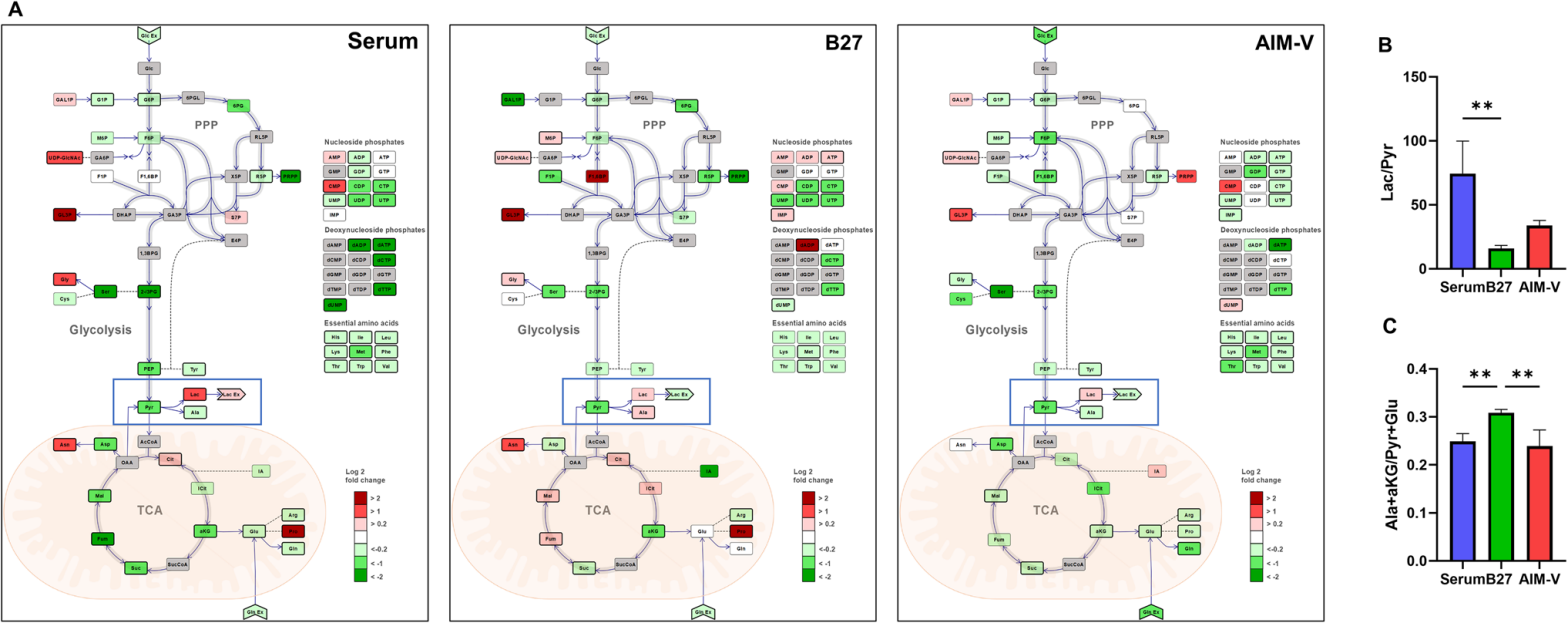
(**A**) Simplified schematic alteration of central carbon metabolism in C2C12 myotubes versus myoblasts with a heat map of the log2 fold change of average metabolites levels in Serum, B27, and AIM-V cultures. The student’s t-test was performed. Bold lines indicate statistically significant metabolite levels (p < 0.05). Dark gray color metabolites were not determined. Direct reactions are shown by continuous lines, a series of reactions are indicated by dashed lines. *TCA* tricarboxylic acid, *PPP* pentose phosphate pathway, *Glc ex* consumption of glucose, *Gln ex* consumption of glutamine, *Lac ex* secretion of lactate. Significant different pyruvate metabolisms were shown in myotubes cultured in Serum, B27, AIM-V at day 7. The graphs were illustrated using OMIX 2.0 software^59^. (**B**) Pyruvate reduction into Lactate and (**C**) Transamination to Alanine. (n) = 4. **p < 0.01. Data presented as average ± standard deviation (SD).

We further compared the intracellular metabolite profiles between Serum, B27, AIM-V medium cultures. Therefore, intracellular metabolic switching from proliferating myoblasts (day 1) to differentiated myotubes (day 7) was illustrated in each medium (Fig. 7A). C2C12 cultured in B27 and Serum, which shared DMEM as a basic medium, commonly had elevated intracellular levels of glycine, proline, asparagine, whilst no change was observed in AIM-V culture. They are non-essential amino acids and are not included in DMEM basic medium except glycine (Supplementary table 3). Interestingly, proliferating myoblasts cultured in AIM-V showed a significantly higher concentration of Pro, Asn, Arg (only against serum) than Serum and B27 cultures (Supplementary Fig. 11 and Table 4). Unfortunately, the complete formulation of AIM-V is not disclosed. In fact, commercial serum-reduced mediums from the same manufactures, such as Advanced DMEM, MEM, and RPMI, are often supplemented with non-essential amino acids, assuming that non-essential amino acids might be supplemented in AIM-V medium, which was confirmed by Thermo fisher company. A consistent observation regarding elevated glycine during human skeletal muscle differentiation has been reported^44^. However, the normalization procedure to present the final intracellular concentration is not standardized and depends on the individual lab. It might be controversial for direct comparison since they reported decreased proline levels during differentiation.

Secondary, the reduction and transamination of pyruvate showed a significant difference between Serum, B27, AIM-V cultures (Fig. 7A). In most cells, the major source of cytosolic pyruvate is the last step of glycolysis. Pyruvate is further reduced into lactate by lactate dehydrogenase (LDH) or transamination conjugated with glutamate takes place to produce alanine and α-ketoglutarate by alanine transaminase (ALT)^45^. Pyruvate reduction was decreased only in B27 cultures (Fig. 7B), resulting in low production of lactate accompanied by increased TCA intermediates levels, suggesting that oxidative metabolism is dominant. In contrast, both Serum and AIM-V cultures showed a high level of intracellular lactate, but excretion of lactate is significantly higher only in Serum culture.

Regarding pyruvate transamination, only myotubes in B27 cultures showed significantly elevated alanine levels compared to Serum and AIM-V cultures (Fig. 7A). Surprisingly, the ratio of the sum of alanine and α-ketoglutarate to pyruvate and glutamate also showed the highest levels in B27 cultures (Fig. 7C). Moreover, a significantly higher absolute concentration of the non-essential amino acids including Ala, Asp, Cys, Gln, Glu, Ser, Tyr, Arg (only against Serum), Asn (only against AIM-V) was observed in myotubes (day 7) cultured in B27 compared to AIM-V and Serum culture (Supplementary Fig. 2 and Table 4). Taken together, non-essential amino acids and the reduction and transamination of pyruvate were shown distinctly different between medium (Serum, B27, AIM-V).

### Intracellular metabolic profiles could be applied to determine muscle phenotypes

**Figure 8:**
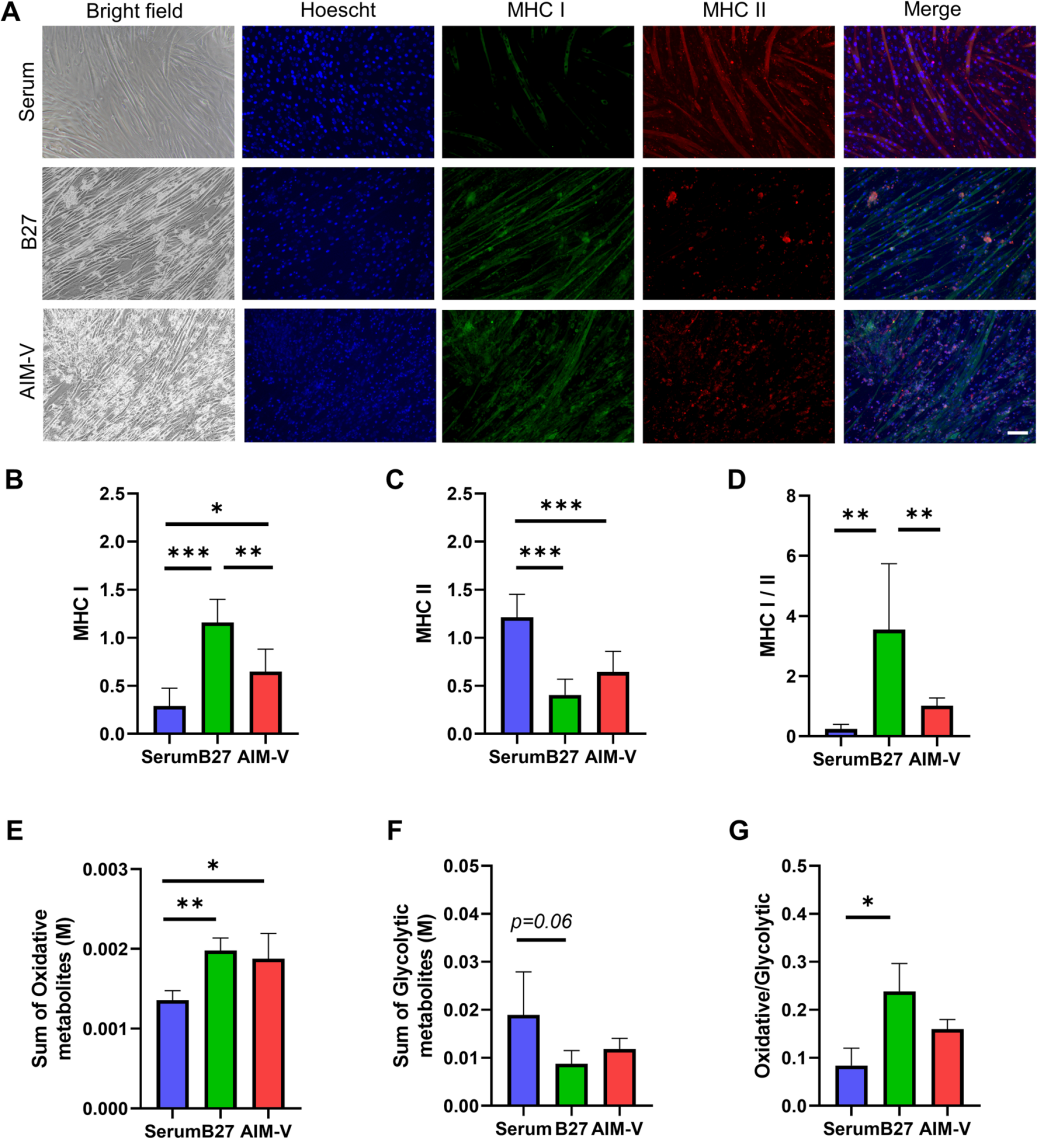
(**A**) Representative images of phase-contrast and immunostaining of MHC I and II in C2C12 cells at day 7 in Serum, B27, and AIM-V cultures. Images show immunostaining for MHC I (green), MHC II (red) and Hoechst counterstained nuclei (blue). Merged panels show composite images. Scale bar indicates 100 µm. Quantification of MHC I and II images from A was shown following figures. The positively MHC I and II—stained area was normalized to nuclei area. (**B**) Quantification results of MHC I (slow type)—positive stained myotubes. (**C**) Quantification of MHC II (fast type)-positive stained myotubes. (**D**) The ratio of quantified MHC I to MHC II, indicating slow type of muscle is more dominant. The experiments were performed three times and representative results are shown. (**E**) The sum of the glycolytic metabolism-related metabolites (G6P, F6P, F1, 6BP, 2/3-PG, PEP, Pyr, and Lac) and (**F**) Oxidative metabolism-related metabolites (Cit, Icit, aKG, Suc, Fum, IA, and Mal). (**G**) The ratio of sum of oxidative to glycolytic-related metabolites. Data presented as average ± standard deviation (SD). (n) = 4. *p < 0.05, **p < 0.01, ***p < 0.001.

To date, there are only very few studies to determine the difference of intracellular metabolite profiles between fast and slow types of muscles. Skeletal muscle phenotype could be determined by the type of myosin heavy chain (MHC) isoform. Fast muscles and slow muscles contain MHC II (type II) and MHC I (type I), respectively^46^. In fact, slow muscles (type I fibers) and fast muscle (type II fibers) rely mainly on oxidative and glycolytic metabolism, respectively^47^. Therefore, we further investigated whether intracellular metabolic profiles could distinguish the muscle phenotype. To investigate the muscle phenotype, immunostaining of MHC type I and II were performed on day 7 of Serum, B27, and AIM-V cultures (Fig. 8A). Serum culture showed mostly MHC II phenotype than MHC I, indicating the fast type of muscle cells are dominant, while B27 culture indicated significantly predominant of MHC I expression than MHC I (Fig. 8B,C). Surprisingly, a similar trend is also shown by calculating the sum of intracellular glycolytic-related metabolites (Fig. 8E) and oxidative-related metabolites (Fig. 8F, Supplementary table 2). In particular, the ratio of slow to the fast type of muscles (MHC I to MHC II) by immunostaining (Fig. 8D) and the ratio of the sum of oxidative to glycolytic metabolite set (Fig. 8G) presented the same trend between Serum, B27, and AIM-V cultures.

Previously, C2C12 myotubes cultured in serum showed high expression of *MYH1* and *MYH4* associated with type II (glycolytic) fibers, indicating fast muscle type^48^. Primary rat myoblast cultured in AIM-V showed intermediate muscle type (sharing slow and fast muscle type), as higher expression of MYH2 compared to the Serum culture was observed^13^. Surprisingly, our metabolite profiling was consistent with a previous study that Serum culture showed the fast type of muscle glycolytic metabolism^48^, confirming that intracellular metabolic data can be used as a tool to assess the phenotype of muscle.

Moreover, lactate was thought to be a waste product of anaerobic glycolysis. However, lactate can be formed through aerobic glycolysis and act as the vehicle between glycolytic and oxidative metabolism. Glycolytic cells act as lactate producers, whereas oxidative cells act as lactate consumers^49^. Therefore, we suspected that myotubes cultured in B27 and Serum act as consumer and producer cells for lactate, suggesting the oxidative and glycolytic types of muscles, respectively. C2C12 cells in AIM-V might share oxidative and glycolytic types, due to the lower secretion even though they showed high intracellular lactate concentrations (Supplementary Fig. 2). However, the current study is limited to measuring the export and uptake rate of intracellular lactate. Therefore, the investigation of the relation between lactate metabolism and muscle phenotypes might be interesting as a future study.

Furthermore, higher levels of non-essential amino acids and total amino acids were observed in the slow type compared to the fast type of muscle^50^. In addition, the levels of intracellular alanine, glutamine, and histidine showed a significant high correlation with the slow type of muscle^51^, which is in line with our result from B27 culture (Supplementary Fig. 2). Therefore, we can confirm that the most dominant muscle type of C2C12 in B27 is the slow type of muscle.

The distinction of muscle types should be considered in the field of artificial meat research. For example, slow types of muscles are dominant in red meat, such as beef, whereas white meat, including mouse and pig, is rich in the fast type of muscles^27^. In addition, the slow type of muscle can offer a more rich flavor and taste due to high levels of alanine, glutamate, and aspartate^52^, suggesting that the development of different muscle fibers must be considered in future artificial meat research. Taken together, C2C12 myotubes cultured in B27, Serum, AIM-V showed the oxidative, glycolytic, mixed metabolic phenotypes of slow, fast, and intermediate muscles, respectively.

## Conclusion

We performed the metabolic analysis of C2C12 myoblasts and myotubes in two different types of serum-free culture models (B27 and AIM-V) and compared them with the conventional serum-supplemented culture model to explore the impact of the serum-free culture on skeletal muscle cells. High cell viability was shown during 1 week of cultivation and myotubes showed different phenotypes in terms of the size and muscle twitching between the different medium cultures. The rate of consumption glucose versus secretion of lactate was increased after myogenic differentiation. Significant upregulated intracellular levels of GL3P and UDP-GlcNAc in myotube were shown in all culture models, which can be applied for the assessment of muscle cells differentiation. Non-essential amino acids and reduction and transamination of pyruvate were different between the medium culture. B27 could be the serum substitute for generating slow and oxidative metabolic phenotype muscle. The metabolic profile of AIM-V culture was more similar compared to serum culture than B27. We believe that this work will suggest that metabolic profiling can be used as a tool for the assessment of muscle cells and the distinction of their phenotypes.

## Methods and materials

### Chemicals and materials

All chemicals and materials were purchased from Sigma Aldrich, unless specifically defined. U^13^C(^15^ N) labeled isotope chemicals were obtained from Cambridge isotope laboratories.

### Cell culture and maintenance

C2C12 murine myoblasts were purchased from the American type culture collection (ATCC CRL-1772, lot number 70024392). Cells were cultured and maintained in tissue culture flask T75 or T175 flasks (Treated, VWR, USA) in growth medium containing high glucose DMEM medium supplemented with 10% Fetal Bovine Serum (FBS) and 1% penicillin–streptomycin (15140122, Thermo Fisher Scientific, USA) in an incubator at 37 °C under a humidified atmosphere of 5% CO_2_ supply (HERAcell 150i, Thermo fisher Scientific, USA). Cells are subcultured when they reached 50 to 60% of confluence. The passage number from 3 to 5 are used in this research.

### Proliferation and differentiation culture in serum-supplemented and serum-free mediums

Before seeding the cells into the plate, all plates should be coated with ECL solution (ECL cell attachment matrix, 08-110). Briefly, ECL solution was prepared following concentration (5 µl of ECL solution/1 ml of DMEM medium), and then 50 µl, 1 ml, 5 ml of ECL solution were placed into the 96 wells, 6 wells, and 90 mm culture plates (Treated for increased cell attachment, VWR, USA). After incubating for 1 to 2 h at room temperature, coated plates were washed with PBS buffer followed by air-drying in the sterile bench. Cells were seeded at 7500 cells/cm^2^ into plates and proliferated for 2 days to reach 90%-100% confluency in 4 different mediums (only DMEM, DMEM + 10% FBS (lot number; BCBV9857, DMEM + 2% B27 (17504044, Thermo Fisher Scientific, USA), AIM-V (A3830801, Thermo Fisher Scientific, USA). The culture mediums were replaced with DMEM, DMEM + 2% horse serum (lot number; 19D017), DMEM + 1% B27, AIM-V, respectively. The fresh medium was replaced every 2 days and cells were incubated 5 days more to stimulate myotube formation. To reduce the experimental variability, the same lot number of serum was supplemented for all the experiments.

### Cell morphology monitoring

Cell morphology was observed under the bright field phase-contrast microscope (Eclipse T_S_2, Nikon, Japan) and the diameter of myotubes was carefully measured using NIS Elements F 4.51 software under 4X objective lens. The myotube twitching was recorded under 10X objective lens using Bandicam software (Bandicam.Inc, Seoul, Korea). Detail method for analyzing muscle twitching video is described in the supplementary material.

### Measurement of cell viability and cell death

Cell viability was measured using PrestoBlue assay (A13262, ThermoFisher, USA). Briefly, cells were seeded in ECM-coated black 96 wells plate and cultivated for 1 week in the different medium according to the above culture method. The PrestoBlue reagent was added at 10% of total volume and incubated for 30 min at 37 °C. The fluorescence was measured at 560 nm for the excitation at 590 nm for emission using a microplate reader machine (Spark 20 M, Tecan, Switzerland) at indicating culture time points.

Cell death was determined by the measurement of released lactate dehydrogenase (LDH) (MAK066) into the medium. The medium was collected and immediately stored at − 80 °C. LDH activity in the collected medium was measured in duplicate according to the manufacturer’s instructions.

### Cell counting for the measurement of growth rate and doubling time

The total cell number at day 0, 1, 2 was counted using Moxi^z^ cell counter machine equipped with type S cassettes (Orflo Technologies). The doubling times (DT) were determined according to the following exponential growth equation regression: DT = T ln2/ln(Xe/Xb); where T is the incubation time, Xb is the cell number at the beginning of the incubation time and Xe is the cell number at the end of the incubation time^53,54^.

### CK (Creatine kinase) activity assay

The cells are washed with PBS buffer once and then lysed with M-PER buffer supplemented with Halt protease and phosphatase inhibitor (100X, 78446, Thermo Scientific, USA). Collected lysates were centrifuged (4 °C, 14000Xg, 10 min) and only supernatants were stored immediately at − 80 °C. To measure total protein content, BCA protein assay kit (23225, Thermo Scientific, USA) was used. CK activity was determined using Creatine Kinase Activity Assay Kit (MAK116) according to the manufacturer's instructions. Briefly, 100 µl of reconstituted reagent was added with 10ul of cell samples in a 96 well microplate. The plate was incubated at 37 °C and measured 20 and 25 min at 340 nm using a microplate reader (Spark 20 M, Tecan, Switzerland), then 600 factor was applied for the calculation of CK activity. The calculated CK activity value was corrected by the total protein content.

### Immunofluorescence

The cells were fixed in 4% paraformaldehyde for 15 min at room temperature and permeabilized with 0.1% triton X-100 in PBS for 15 min. After washing with PBS buffer three times, cells were blocked in 1% BSA in TBST solution for 1 h and then incubated with diluted MHC primary antibody (Rabbit anti-MHC class I, ab281901, Abcam, 1:100, and mouse anti-MHC class II, ab55152, Abcam, 1:100) at 4˚C for overnight. After washing with PBS, cells were incubated second antibodies conjugated with Alexa Fluor 488 and 594. (Goat anti rabbit Alexa Fluor 488, ab150081, 1:500 and Goat anti-mouse Alexa Fluor 594, A-11032, Thermo Fisher Scientific, 1:200) for 1 h at room temperature. 1 µg/ml of Hoechst 33342 was used for the staining of nuclei acid. All images were taken using a fluorescent microscope (Nikon Eclipse Ts2) equipped with 10× objective lens (Plan Fluor, Nikon) and image software (NIS element version 4.51.00, Nikon). For the image analysis, 2–3 randomly selected fields were used from three independent experiments. Evaluation of fluorescence intensity was carried out with ImageJ FIJI software^55^.

### Preparation of intracellular metabolites extraction procedure from cells and medium

Cells were seeded into ECM-coated 90 mm culture plates and 6 well plates for the metabolic sampling and the measurement of cell density and the average of cell volume. The cells were collected on day 1 and 7. Sampling was performed according to the previous publication with slight modification^22^. In detail, each cell plate was placed on the ice pack. After discarding the cell medium, cells were quickly washed in 10 ml of cold 0.9% NaCl solution followed by cold 10 ml of MQ-H_2_O. Finally, cells were detached mechanically using by cell scraper in 5 ml of cold MQ:ACN (1:1, v/v) solution and cell solution was transferred in the 50 ml centrifugation tube. This step was repeated once more. Total 10 ml of cell solution in MQ:ACN (1:1, v/v) was immediately quenched in the LN_2_ and stored at − 80 °C. In addition, cell density (cells/ml) and cell volume(pL) were measured from a 6 wells plate, using a Moxi^z^ cell counter machine equipped with type S cassettes (Orflo Technologies, USA).

### Preparation of intracellular metabolites extracts

Intracellular metabolites were extracted from the quenched cell suspension based on the repeated freeze-thawing cycle between LN_2_ and cold water (< 4 °C). Total three repeated freeze–thaw cylces were performed for the disruption of cell membrane by ice crystal formation. Extracted intracellular metabolites solutions were centrifuged (4 °C, 4500xg, 10 min) to remove cell debris. Only 9 ml of extracted solution was collected and immediately quenched in LN2 and lyophilized. Concentrated lyophilized intracellular metabolites extracts can be stored at − 80 °C before analysis. For further analysis, extracts were reconstituted in 500 µl of cold MQ-H_2_O and filtered through spin-filter 3KD cutoff by centrifugation (4 °C, 14000xg, 20 min) and stored at − 80 °C for MS-based metabolites analysis.

### The measurement of extracellular metabolites

Extracellular glucose, lactate, and glutamine were measured from culture medium according to previous publication^56^. Briefly, 2.5 ml of the medium was collected for day 0–1 (day 1) and day 6–7 (day 7) from 90 mm dish culture, respectively. The cell counting was performed using a Moxi^z^ cell counter machine immediately after collecting the medium. After brief centrifugation (4 °C, 1000 rpm, 5 min), the 2 ml of the medium was immediately quenched in LN2 and stored at − 20 °C. After freeze-drying, it was reconstituted in 600 µl of deuterium oxide (d_2_O) and measured by NMR (Bruker Ascent 400 MHz) based on “N PROF_1H” method. 70 mM of creatine solution was used as a quantification reference.

### Targeted MS-based metabolite profiling

Phosphorylated metabolites and TCA intermediates were quantified based on previous publications using by capillary ion chromatography (CapIC)—triple quadrupole mass spectrometer (MS/MS) (Xevo TQ-XS, Waters, USA)^57^. Organic acids and amino acids analysis were performed according to previous publication^22^. Briefly, cell extracts and standards were derivatized using o-benzylhydroxyl amine and phenyl isothiocyanat for organic acids and amino acids analysis, respectively. UPLC (Accunity I-class UPLC, Waters, USA) coupled with TQ-XS was used for the quantification and further instrumental parameters were described in a previous publication^22^.

### Data processing

TopSpin v4.1.1 (Bruker, USA) was used for the data processing of all NMR-spectra to quantify extracellular glucose, lactate, and glutamine in the medium. All MS-based data processing was performed in the TargetLynx application manager of Masslynx 4.1 (Waters Corporation, USA). All responses factors are corrected the corresponding U^13^C(^15^ N)-isotopologue. Intracellular concentration was further corrected for dilution factors and concentrations during sample preparation and normalized to cell density and average cell volumes.

### Statistics and graphical tools

All results are expressed as the average ± standard deviation (SD). To perform the multivariate analysis in Metaboanalyst^58^, all data were normalized with auto-scaling before multivariate statistical tests. The student’s t-test was performed to compare the average of the two groups. One-way ANOVA followed by Tukey HSD was applied for the posthoc analysis using SPSS version 27 for the comparison of multiple groups.

## Supplementary Information

Supplementary Information 1.

Supplementary Video S1.

Supplementary Video S2.

Supplementary Video S3.

## References

[1] Price PJ (2017). Best practices for media selection for mammalian cells. In Vitro Cell. Dev. Biol. Anim..

[2] Brindley DA (2012). Peak serum: Implications of serum supply for cell therapy manufacturing. Regen. Med..

[3] Ben-Arye T, Levenberg S (2019). Tissue engineering for clean meat production. Front. Sustain. Food Syst..

[4] van der Valk J (2010). Optimization of chemically defined cell culture media—Replacing fetal bovine serum in mammalian in vitro methods. Toxicol. In Vitro.

[5] Liu CH, Liu YX, Wu WC (2018). Facile development of medium optimization for antibody production: Implementation in spinner flask and hollow fiber reactor. Cytotechnology.

[6] McGillicuddy N, Floris P, Albrecht S, Bones J (2018). Examining the sources of variability in cell culture media used for biopharmaceutical production. Biotechnol. Lett..

[7] Karnieli O (2017). A consensus introduction to serum replacements and serum-free media for cellular therapies. Cytotherapy.

[8] Orellana N (2020). A new edible film to produce in vitro meat. Foods.

[9] MacQueen LA (2019). Muscle tissue engineering in fibrous gelatin: implications for meat analogs. npj Sci. Food.

[10] Zhang G (2020). Challenges and possibilities for bio-manufacturing cultured meat. Trends Food Sci. Technol..

[11] Kolkmann AM, Post MJ, Rutjens MAM, van Essen ALM, Moutsatsou P (2020). Serum-free media for the growth of primary bovine myoblasts. Cytotechnology.

[12] Will K, Kuzinski J, Palin M-F, Hildebrandt J-P, Rehfeldt C (2014). A second look at leptin and adiponectin actions on the growth of primary porcine myoblasts under serum-free conditions. Arch. Anim. Breed..

[13] Cai A (2018). Myogenic differentiation of primary myoblasts and mesenchymal stromal cells under serum-free conditions on PCL-collagen I-nanoscaffolds. BMC Biotechnol..

[14] Bodiou V, Moutsatsou P, Post MJ (2020). Microcarriers for upscaling cultured meat production. Front. Nutr..

[15] Lawson MA, Purslow PP (2000). Differentiation of myoblasts in serum-free media: Effects of modified media are cell line-specific. Cells Tissues Organs.

[16] Molnar P, Wang W, Natarajan A, Rumsey JW, Hickman JJ (2007). Photolithographic patterning of C2C12 myotubes using vitronectin as growth substrate in serum-free medium. Biotechnol. Prog..

[17] Fujita H, Endo A, Shimizu K, Nagamori E (2010). Evaluation of serum-free differentiation conditions for C2C12 myoblast cells assessed as to active tension generation capability. Biotechnol. Bioeng..

[18] Guo G (2016). Serum-based culture conditions provoke gene expression variability in mouse embryonic stem cells as revealed by single-cell analysis. Cell Rep..

[19] Luhur A (2020). Adapting drosophila melanogaster cell lines to serum-free culture conditions. G3 Genes Genomes Genet..

[20] Guijas C, Montenegro-Burke JR, Warth B, Spilker ME, Siuzdak G (2018). Metabolomics activity screening for identifying metabolites that modulate phenotype. Nat. Biotechnol..

[21] Muroya S, Ueda S, Komatsu T, Miyakawa T, Ertbjerg P (2020). Meatabolomics: Muscle and meat metabolomics in domestic animals. Metabolites.

[22] Røst LM (2020). Absolute quantification of the central carbon metabolome in eight commonly applied prokaryotic and eukaryotic model systems. Metabolites.

[23] Kumar K, Venkatraman V, Bruheim P (2021). Adaptation of central metabolite pools to variations in growth rate and cultivation conditions in *Saccharomyces cerevisiae*. Microb. Cell Factories.

[24] Huang B (2021). Mdfi promotes C2C12 cell differentiation and positively modulates fast-to-slow-twitch muscle fiber transformation. Front. Cell Dev. Biol..

[25] Daskalaki E, Pillon NJ, Krook A, Wheelock CE, Checa A (2018). The influence of culture media upon observed cell secretome metabolite profiles: The balance between cell viability and data interpretability. Anal. Chim. Acta.

[26] Hwang SY (2015). Folic acid promotes the myogenic differentiation of C2C12 murine myoblasts through the Akt signaling pathway. Int. J. Mol. Med..

[27] Listrat A (2016). How muscle structure and composition influence meat and flesh quality. Sci. World J..

[28] Gupta SK (2017). Metabolic engineering of CHO cells for the development of a robust protein production platform. PLoS ONE.

[29] la Fuente IMD (2014). On the dynamics of the adenylate energy system: Homeorhesis vs homeostasis. PLoS ONE.

[30] Levitt DE, Chalapati N, Prendergast MJ, Simon L, Molina PE (2020). Ethanol-impaired myogenic differentiation is associated with decreased myoblast glycolytic function. Alcohol. Clin. Exp. Res..

[31] Ryall JG (2013). Metabolic reprogramming as a novel regulator of skeletal muscle development and regeneration. FEBS J..

[32] Fortini P, Iorio E, Dogliotti E, Isidoro C (2016). Coordinated metabolic changes and modulation of autophagy during myogenesis. Front. Physiol..

[33] Tomczak KK (2004). Expression profiling and identification of novel genes involved in myogenic differentiation. FASEB J..

[34] Fortini P (2016). The fine tuning of metabolism, autophagy and differentiation during in vitro myogenesis. Cell Death Dis..

[35] Homolak J (2021). Is galactose a hormetic sugar? Evidence from rat hippocampal redox regulatory network. bioRxiv.

[36] Luo S (2021). Comparative sensitivity of proliferative and differentiated intestinal epithelial cells to the food contaminant, deoxynivalenol. Environ. Pollut..

[37] Mugabo Y (2016). Identification of a mammalian glycerol-3-phosphate phosphatase: Role in metabolism and signaling in pancreatic β-cells and hepatocytes. Proc. Natl. Acad. Sci. USA.

[38] Kubišta V (1957). Accumulation of a stable phosphorus compound in glycolysing insect muscle. Nature.

[39] Liu X (2018). Mitochondrial glycerol 3-phosphate dehydrogenase promotes skeletal muscle regeneration. EMBO Mol. Med..

[40] Lambert M, Bastide B, Cieniewski-Bernard C (2018). Involvement of O-GlcNAcylation in the skeletal muscle physiology and physiopathology: Focus on muscle metabolism. Front. Endocrinol..

[41] Shi H (2018). Skeletal muscle O-GlcNAc transferase is important for muscle energy homeostasis and whole-body insulin sensitivity. Mol. Metab..

[42] Ogawa M (2012). Terminal differentiation program of skeletal myogenesis is negatively regulated by O-GlcNAc glycosylation. Biochim. Biophys. Acta Gen. Subj..

[43] Zumbaugh, M. D. *Signaling Pathways Regulating Skeletal Muscle Metabolism and Growth* (2021).

[44] Kumar A (2020). Metabolomic analysis of primary human skeletal muscle cells during myogenic progression. Sci. Rep..

[45] Gray LR, Tompkins SC, Taylor EB (2014). Regulation of pyruvate metabolism and human disease. Cell. Mol. Life Sci..

[46] Hyatt JPK (2016). Muscle-specific myosin heavy chain shifts in response to a long-term high fat/high sugar diet and resveratrol treatment in nonhuman primates. Front. Physiol..

[47] Talbot J, Maves L (2016). Skeletal muscle fiber type: using insights from muscle developmental biology to dissect targets for susceptibility and resistance to muscle disease. Wiley Interdiscip. Rev. Dev. Biol..

[48] Abdelmoez AM (2020). Comparative profiling of skeletal muscle models reveals heterogeneity of transcriptome and metabolism. Am. J. Physiol. Cell Physiol..

[49] Brooks GA (2018). The science and translation of lactate shuttle theory. Cell Metab..

[50] Holeček M, Mičuda S (2017). Amino acid concentrations and protein metabolism of two types of rat skeletal muscle in postprandial state and after brief starvation. Physiol. Res.

[51] Mashima D (2019). Correlation between skeletal muscle fiber type and free amino acid levels in Japanese Black steers. Anim. Sci. J..

[52] Komiya Y (2020). Correlation between skeletal muscle fiber type and responses of a taste sensing system in various beef samples. Anim. Sci. J..

[53] Roth, V. Doubling Time—Online computing with 2 points. *Doubling Time Computing* https://www.doubling-time.com/compute.php (2006).

[54] Torres-Guzmán R (2017). Preclinical characterization of abemaciclib in hormone receptor positive breast cancer. Oncotarget.

[55] Schindelin J (2012). Fiji: An open-source platform for biological-image analysis. Nat. Methods.

[56] Søgaard CK (2018). “Two hits—one stone”; increased efficacy of cisplatin-based therapies by targeting PCNA’s role in both DNA repair and cellular signaling. Oncotarget.

[57] Stafsnes MH, Røst LM, Bruheim P (2018). Improved phosphometabolome profiling applying isotope dilution strategy and capillary ion chromatography-tandem mass spectrometry. J. Chromatogr. B Anal. Technol. Biomed. Life Sci..

[58] MetaboAnalyst. https://www.metaboanalyst.ca/.

[59] Droste P, Miebach S, Niedenführ S, Wiechert W, Nöh K (2011). Visualizing multi-omics data in metabolic networks with the software Omix—A case study. Biosystems.

