# Supplementary material for: Serum-free cultures of C2C12 cells show different muscle phenotypes which can be estimated by metabolic profiling
Source: Sci Rep. 2022 Jan 17;12:827. doi: 10.1038/s41598-022-04804-z (PMC8764040; doi:10.1038/s41598-022-04804-z)
Supplement: Supplementary file 1 — Supplementary Information 1. [file 41598_2022_4804_MOESM1_ESM.docx]

**Title :**

**Serum-free cultures of C2C12 cells show different muscle phenotypes which can be estimated by metabolic profiling**

**Full name of authors** :

Mi Jang^1^, Jana Scheffold^1^, Lisa Marie Røst^1^, Hyejeong Cheon^2^, Per Bruheim^1^.

**Affiliation of authors**:

^1.^ Department of Biotechnology and Food Science, Norwegian University of Science and Technology, Hogskoleringen 1, 7491 Trondheim, Norway.

^2.^ PoreLab, Department of Physics, Norwegian University of Science and Technology, Hogskoleringen 1, 7491 Trondheim, Norway.

**Corresponding author** : Per Bruheim

Address: Department of Biotechnology and Food Science, Norwegian University of Science and Technology, Hogskoleringen 1, 7491 Trondheim, Norway.

**e-mail : per.bruheim@ntnu.no**

**Supplementary table 1**. List of metabolite abbreviations

| **Class** | **Abbreviation** | **Full name** |
| --- | --- | --- |
| Glycolysis | G6P | Glucose-6-phosphate |
|  | F6P | Fructose-6-phosphate |
|  | F1,6BP | Fructose 1,6-biphosphate |
|  | 2/3-PG | 2-/3-Phopho-D.glycerate |
|  | PEP | Phosphoenolpyruvic acid |
|  | Pyr | Pyruvic acid |
| Lac | Lac | Lactate |
| PPP | 6PG | 6-Phospho-D-Gluconate |
|  | PRPP | Phosphoribosyl pyrophosphate |
|  | R5P | D-Ribose-5-phosphate |
|  | S7P | D-Sedoheptulose-7-phosphate |
| Other sugar phosphates | F1P | Fructose-1-phosphate |
|  | G1P | Glucose-1-phosphate |
|  | GAL1P | Galactose-1-phosphate |
|  | GL3P | Glycerol-3-phosphate |
|  | M6P | mannose-6-phosphate |
|  | UDP-GlcNAc | Uridine diphosphate *N*-acetylglucosamine |
| TCA cycle | Cit | Citrate |
|  | Icit | Isocitrate |
|  | aKG | α-ketoglutarate |
|  | Suc | Succinate |
|  | Fum | Fumarate |
|  | IA | Itaconic acid |
|  | Mal | Malate |
| Nucleoside phosphates | AMP | Adenosine monophosphate |
|  | ADP | Adenosine diphosphate |
|  | ATP | Adenosine triphosphate |
|  | CMP | Cytidine monophosphate |
|  | CDP | Cytidine diphosphate |
|  | CTP | Cytidine triphosphate |
|  | GMP | Guanosine monophosphate |
|  | GDP | Guanosine diphosphate |
|  | GTP | Guanosine triphosphate |
|  | IMP | Inosine monophosphate |
|  | UMP | Uridine monophosphate |
|  | UDP | Uridine diphosphate |
|  | UTP | Uridine triphosphate |
| Deoxy nucleoside phosphates | dADP | Deoxyadenosine diphosphate |
|  | dATP | Deoxyadenosine triphosphate |
|  | dCTP | Deoxycytidine triphosphate |
|  | dTDP | Deoxythymidine diphosphate |
|  | dTTP | Deoxythymidine triphosphate |
|  | dUMP | Deoxyuridine monophosphate |
| Amino acids | Ala | Alanine |
|  | Arg | Arginine |
|  | Asn | Asparagine |
|  | Asp | Aspartate |
|  | Cys | Cysteine |
|  | Gln | Glutamine |
|  | Glu | Glutamate |
|  | Gly | Glycine |
|  | His | Histidine |
|  | Ile | Isoleucine |
|  | Leu | Leucine |
|  | Lys | Lysine |
|  | Met | Methionine |
|  | Phe | Phenylalanine |
|  | Pro | Proline |
|  | Ser | Serine |
|  | Thr | Threonine |
|  | Trp | Tryptophan |
|  | Tyr | Tyrosine |
|  | Val | Valine |

**Supplementary figure 1**. Heat map of normalized intracellular metabolites (Auto scale) pools in myoblasts (day 1) cultured in 3 different medium conditions (Serum *vs.* B27 *vs.* AIM-V). The graph was illustrated using Metaboanalyst 5.0.


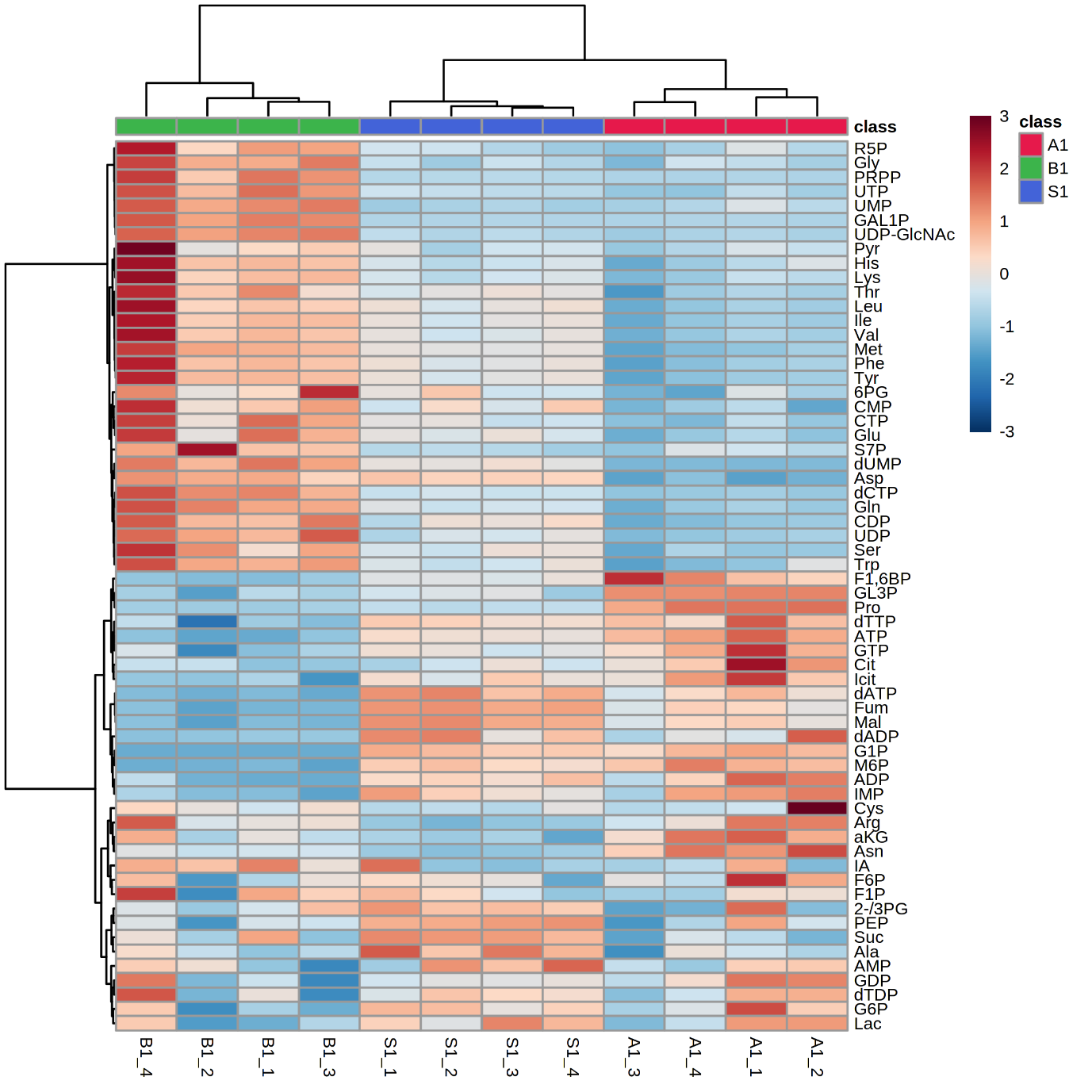


**Supplementary figure 2**. Heat map of normalized intracellular metabolites (Auto scale) pools in myotubes (day 7) cultured in 3 different medium conditions (Serum *vs.* B27 *vs*. AIM-V). The graph was illustrated using Metaboanalyst 5.0.


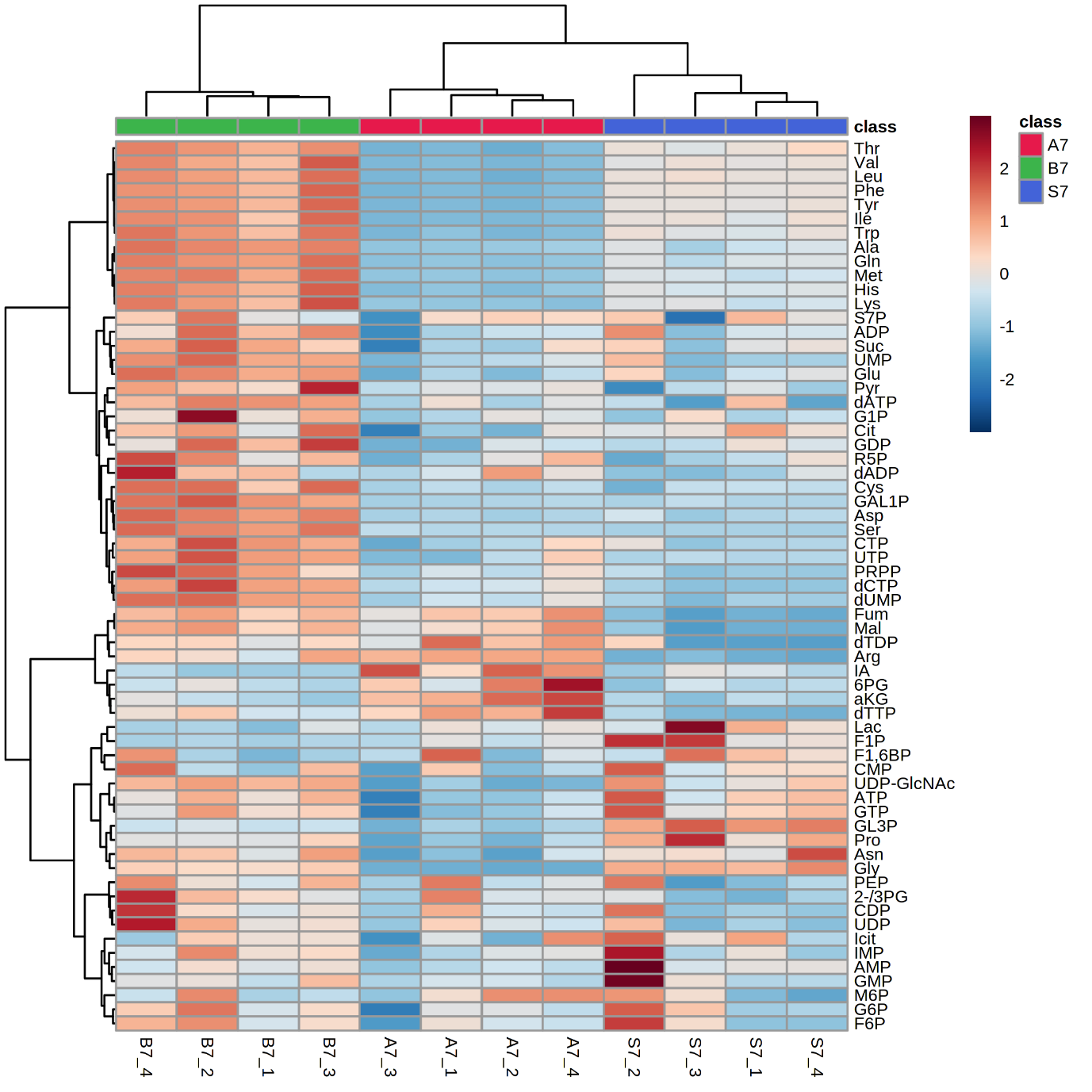


**Supplementary figure 3**. Average of Adenyl Energy charge in myoblasts (day 1) and myotubes (day 7) in DMEM, Serum, B27, AIM-V. (n) =4. Data presented as average ± standard deviation (SD).

**Supplementary figure 4**. Video analysis of myotubes twitching (A) Scheme of the process step of video analysis to present the muscle fiber contraction. (B) The visualized muscle cells beating signal for 20 seconds in Serum, B27, and AIM-V culture at day 7. The red box shows the magnified images from indicating a location for 1.5 seconds. A red arrow indicates time to single muscle twitching movement. (C) Contour maps of myotube twitching. All experiments were performed 3 times and representative results are presented.


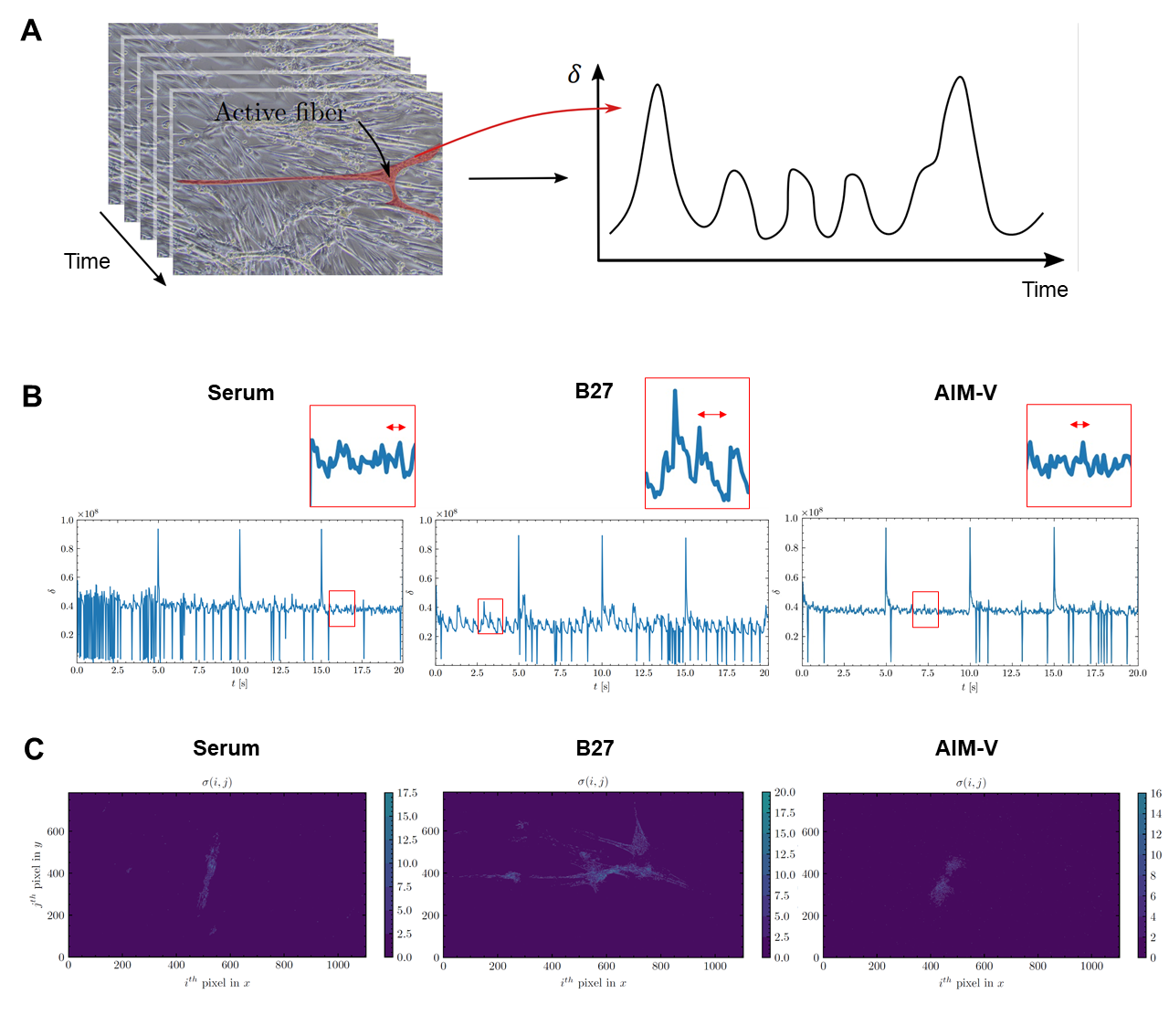


**Supplementary table 2**. The sum of the glycolytic metabolism-related metabolites (G6P, F6P, F1,6BP, 2/3-PG, PEP, Pyr, and Lac) and Oxidative metabolism-related metabolites (Cit, Icit, aKG, Suc, Fum, IA, and Mal). The ratio of sum of glycolytic to Oxidative-related metabolites. Data presented as average ± standard deviation (SD). **p*<0.05, ***p*<0.01 *vs.* Serum

|  | **Serum** | **B27** | **AIM-V** |
| --- | --- | --- | --- |
| Sum of Glycolytic- related metabolites (M) | 0.0189±0.0077 | 0.0088±0.0024 *(P=0.06, vs.Serum)* | 0.0119±0.0019 |
| Sum of Oxidative-related metabolites (M) | 0.0013±0.0001 | 0.0019±0.0001** | 0.0019±0.0003* |
| Ratio of  Oxidative/Glycolytic | 0.084±0.031 | 0.239±0.05* | 0.16±0.018 |

**Supplementary table 3.** The composition and concentrations of DMEM medium.

| **Component** | **g/L** |
| --- | --- |
| **Inorganic Salts** |  |
| CaCl_2_ | 0.2 |
| Fe(NO_3_)_3_ • 9H2O | 0.0001 |
| MgSO_4_ | 0.0976 |
| KCl | 0.4 |
| NaHCO_3_ | 3.7 |
| NaCl | 6.4 |
| NaH_2_PO_4_ | 0.109 |
| **Amino Acids** |  |
| L-Arginine • HCl | 0.084 |
| L-Cystine • 2HCl | 0.0626 |
| L-Glutamine | 0.584 |
| Glycine | 0.03 |
| L-Histidine • HCl • H_2_O | 0.042 |
| L-Isoleucine | 0.105 |
| L-leucine | 0.105 |
| L-Lysine • HCl | 0.146 |
| L-Methionine | 0.03 |
| L-Phenylalanine | 0.066 |
| L-Serine | 0.042 |
| L-Threonine | 0.095 |
| L-Tryptophan | 0.016 |
| L-Tyrosine • 2Na • 2H_2_O | 0.10379 |
| L-Valine | 0.094 |
| **Vitamins** |  |
| Choline Chloride | 0.004 |
| Folic Acid | 0.004 |
| myo-Inositol | 0.0072 |
| Niacinamide | 0.004 |
| D-Pantothenic Acid • ½Ca | 0.004 |
| Pyridoxine • HCl | 0.004 |
| Riboflavin | 0.0004 |
| Thiamine • HCl | 0.004 |
| **Other** |  |
| D-Glucose | 4.5 |
| Phenol Red • Na | 0.0159 |
| Pyruvic Acid • Na | 0.11 |

**Supplementary table 4.** Amino acid profiles on day 1 (Myoblast) and day 7 (Myotube). The ratio of Serum *versus* B27, Serum *versus* AIM-V, and B27 *versus* AIM-V cultures. Data presented as average ± standard deviation (SD). *p<0.05. AA indicates amino acids.

|  | **Myoblast (Day 1)** | | | **Myotube (Day 7)** | | |
| --- | --- | --- | --- | --- | --- | --- |
| **Name of AA** | **Serum/**  **B27** | **Serum/**  **AIM-V** | **B27/**  **AIM-V** | **Serum/**  **B27** | **Serum/**  **AIM-V** | **B27/**  **AIM-V** |
| Sum of AA | 0.737398 | 1.219981 | 1.654441 | 0.746095* | 2.25001* | 3.015715* |
| Ala | 1.214782* | 1.255867* | 1.03382 | 0.512225* | 1.434698* | 2.800914* |
| Arg | 0.516064 | 0.478402* | 0.927022 | 0.504432* | 0.422794* | 0.838159 |
| Asn | 0.671063* | 0.381435* | 0.568405* | 0.994121 | 1.286288 | 1.293895* |
| Asp | 0.89644 | 2.04648* | 2.282897* | 0.288781* | 1.186267 | 4.107846* |
| Cys | 0.603306 | 0.472413 | 0.783042 | 0.470445* | 0.976586 | 2.075879* |
| Gln | 0.639259* | 1.32238* | 2.068614* | 0.489528* | 2.021878* | 4.13026* |
| Glu | 0.81156* | 1.202704 | 1.481967* | 0.64921* | 1.284669 | 1.978819* |
| Gly | 0.703219* | 1.024809 | 1.457311* | 1.254449* | 5.801957* | 4.625105* |
| His | 0.648041* | 1.167033 | 1.800863* | 0.616711* | 1.570327* | 2.546292* |
| Ile | 0.656791* | 1.700426 | 2.588989* | 0.645983* | 2.235268* | 3.460259* |
| Leu | 0.694562 | 1.718571 | 2.474322* | 0.672597* | 2.309502* | 3.43371* |
| Lys | 0.523521* | 1.356475 | 2.591062* | 0.578441* | 1.587699* | 2.744789* |
| Met | 0.683677* | 1.6859* | 2.46593* | 0.505887* | 1.731814* | 3.423324* |
| Phe | 0.700297* | 1.586557 | 2.265549* | 0.660369* | 2.157116* | 3.266529* |
| Pro | 1.852728 | 0.263025* | 0.141966* | 1.262562 | 1.766896* | 1.399453* |
| Ser | 0.782753* | 1.230689 | 1.572258* | 0.10049* | 0.598868 | 5.959457* |
| Thr | 0.721604* | 1.435767 | 1.989687* | 0.681692* | 2.294734* | 3.36623* |
| Trp | 0.62686* | 1.404222 | 2.240088* | 0.632579* | 1.997269* | 3.157342* |
| Tyr | 0.68162* | 1.638371* | 2.403642* | 0.645859* | 2.158255* | 3.34168* |
| Val | 0.628297* | 1.604662 | 2.553987* | 0.646823* | 2.134218* | 3.29954* |

**Supplementary material and methods**

**Muscle contraction signal analysis**

The pulse of muscle contraction signals was processed by analyzing a set of frame images extracted from a video based on previous publications with slightly modified equation^1,2^. A 784*×1104* matrix at *k*^th^ frame image is extracted from a sample video when *k* is from 1 to 600. The playtime of each sample video was 20 seconds and FPS (Frame Per Second) was set to 30 ($20\times30=600$ frames). The *784×1104* matrix is from a frame image with 784 pixels in height and 1104 pixels in width. Here, an index that shows the degree of activation at each frame, $\delta_{k}$, is described as:

$$\delta_{k}=\sum_{i}^{m} \sum_{j}^{n} \sqrt{{{(M_{k+1}[i,j]-M}_{k}[i,j])}^{2}} where k\in\{1,2, ...,N-1\}, m=784, n=1104.$$

where *i* and *j* denote the row index and the column index, respectively, and N=600, the total number of the frames.

We further showed muscle movements as a contour map by introducing the standard deviation of $\delta$[*i, j*], [*i, j*]. Here, [*i, j]* stands for the position of a single-pixel such as *i*^th^ pixel in the x-direction and *j*^th^ pixel in the y-direction. $\delta_{k}[$*i, j*], 𝛿[*i, j*] at the *k*th frame matrix, is rewritten as:

$\delta_{k}[i,j]=\sqrt{{{(M_{k+1}[i,j]-M}_{k}[i,j])}^{2}} where k\in\{1,2, ...,N-1\}, i\in\{1,2, ...,784\}, j\in\{1,2, ...,1104\}$,

therefore,

$$\delta_{k}=\sum_{i}^{m} \sum_{j}^{n} \delta_{k}[i,j].$$

The standard deviation of [*i, j*] for totally *N* frame matrices is described as:

$$\sigma[i,j]=\sqrt{\sum_{k}^{N} \frac{\left( \delta_{k}[i,j]-\delta_{avg}[i,j] \right)^{2}}{N}}$$

where $\delta_{avg}$[*i, j*] is the average of N number of $\delta[i, j]$. Therefore, [*i, j*] indicates the degree of active movement where the muscle contraction and expansion are active.

**References**

1. Maddah, M. *et al.* A Non-invasive Platform for Functional Characterization of Stem-Cell-Derived Cardiomyocytes with Applications in Cardiotoxicity Testing. *Stem Cell Reports* **4**, 621–631 (2015).

2. Polina Golland, N. H. C. B. J. H. and R. H. eds. *LNCS 8674 - Medical Image Computing and Computer-Assisted Intervention – MICCAI 2014*. *Proceedings, Part I Medical Image Computing and Computer-Assisted Intervention-MICCAI* (2014).

**Supplementary video 1.** Twitching of C2C12 myotubes cultured in AIM-V.

**Supplementary video 2.** Twitching of C2C12 myotubes cultured in B27.

**Supplementary video 3.** Twitching of C2C12 myotubes cultured in Serum.
